# White matter microstructure of language pathways in non-verbal autism: insights from diffusion tensor imaging and myelin water imaging

**DOI:** 10.3389/fnhum.2025.1551868

**Published:** 2025-08-22

**Authors:** Dominika Slušná, Jordi Muchart-López, Wolfram Hinzen, Erick J. Canales-Rodríguez

**Affiliations:** ^1^Department of Translation and Language Sciences, Pompeu Fabra University, Barcelona, Spain; ^2^Hospital Sant Joan de Déu, Barcelona, Spain; ^3^Institució Catalana de Recerca I Estudis Avancats (ICREA), Barcelona, Spain; ^4^Signal Processing Laboratory (LTS5), École Polytechnique Féderale de Lausanne (EPFL), Lausanne, Switzerland

**Keywords:** non-verbal autism, absent speech, white matter tracts, microstructure, myelin water imaging, diffusion tensor imaging

## Abstract

**Introduction:**

Absence of language development is a condition encountered across a large range of neurodevelopmental disorders, including a significant proportion of children with autism spectrum disorder. The neurobiological underpinnings of non-verbal ASD (nvASD) remain poorly understood.

**Methods:**

This study employed multimodal MRI to investigate white matter (WM) microstructural abnormalities in nvASD, focusing on language-related pathways. We analyzed diffusion tensor imaging (DTI) metrics–fractional anisotropy (FA), mean diffusivity (MD), and radial diffusivity (RD)–alongside myelin water imaging (MWI) metrics, including myelin water fraction (MWF) and intra/extracellular water T2 relaxation time (T2IE). A cohort of 10 children with nvASD and 10 age-matched typically developing controls was examined across eight major language-related tracts and the corticospinal tract (CST) as a motor reference.

**Results:**

While DTI and MWI metrics showed no significant inter-group lateralization differences, MWF and T2IE exhibited pronounced lateralization exclusively in the nvASD group. Results also revealed significant microstructural differences in nvASD. MD and RD were the most sensitive DTI parameters, demonstrating widespread increases, whereas FA was less discriminatory. MWF exhibited the largest percentage change relative to controls (25–50%), suggesting a marked reduction in myelin content within affected tracts. Concurrently, widespread increases in T2IE indicate a less densely packed extra-axonal space, consistent with altered axonal integrity and reduced cellular surface area per unit volume.

**Discussion:**

These findings align with prior evidence linking myelin abnormalities to ASD. Notably, microstructural differences were not restricted to language-related tracts but also extended to the CST, suggesting a more extensive WM disruption in nvASD. The absence of significant correlations between MRI-derived metrics and clinical measures highlights the complexity of the neurobiological alterations in nvASD. As the observed lateralization patterns may reflect, in part, the influence of methodological variability in tract definition, segmentation strategy, and tractography method, these results should be interpreted with caution. Future studies with larger cohorts and longitudinal designs are required to clarify the developmental trajectory of these microstructural abnormalities, their relationship with language impairment severity, and their potential role as biomarkers for nvASD.

## 1 Introduction

Absence of language development, as encountered across a large range of neurogenetic disorders, is an important window into the neural basis of language. Lack of phrase speech by school age also affects a considerable proportion of individuals with autism spectrum disorders (ASD) (Norrelgen et al., 2015). Along with a failure to compensate through other expressive language modalities, such as writing or sign language, children with non-verbal ASD (nvASD) show proportionally low language comprehension (Slušná et al., 2021). This language profile coexists with an absence of broad motor difficulties and is established despite optimal exposure to learning opportunities and interventions over the preschool years, including in a minority with non-verbal IQ in the normal range (Tager-Flusberg and Kasari, 2013). There is variability in the level of single-word speech production, if any, that children with nvASD eventually attain, and this variability is strongly linked to the underlying language comprehension. There is little evidence at present that the comprehension and production profile changes considerably over the course of a lifetime (Slušná et al., 2021).

The neural signature of such severe developmental dysfunction remains practically uncharted, yet is likely to harbor invaluable insights into the neurobiological mechanisms of language. Complex brain function is thought to rely on the integration of information among distributed brain areas, subserved by white matter (WM) connections, which represent the structural backbone for functional circuitry (de Schotten and Forkel, 2022). While this structural backbone is macroscopically in place at birth, it undergoes dramatic microstructural modulation during the first years of life, in tandem with developing cognitive abilities (Hagmann et al., 2010; Bells et al., 2019; Dai et al., 2019), including receptive and expressive vocabulary growth (O’Muircheartaigh et al., 2013; Corrigan et al., 2022). Probing the microstructural integrity of the language-related WM connectome in its dorsal (speech-production-related) and ventral (speech-comprehension-related) routes constitutes a crucial step toward identifying neural markers of the phenotype of absent speech and was the aim of the present study, which was focused on the instance of nvASD.

Alterations in WM development have been recognized as common to a variety of neurodevelopmental disorders (Fields, 2008; Swanson and Hazlett, 2019). In ASD, a recent meta-analysis of studies mostly of high-functioning ASD (Li et al., 2022) established a significantly altered microstructure across the dorsal and ventral language-related WM pathways, as indicated by diffusion tensor imaging (DTI) metrics. A decrease in fractional anisotropy (FA) and an increase in mean diffusivity (MD) were also observed, more prominently in left-hemispheric tracts (Li et al., 2022). Very few studies are available on nvASD. An early study (Wan et al., 2012) reported a reversed laterality of the arcuate fasciculus (AF) volume in five nvASD cases. This finding has not been corroborated in a larger nvASD sample (Olivé et al., 2022), which showed no effect on AF volume or left-hemispheric FA laterality but found a decrease in FA in a ventral pathway—the inferior fronto-occipital fasciculus (IFOF)—in nvASD compared to healthy controls (HC), suggesting difficulties with language meaning in this group. In addition, FA-indexed microstructural maturation of the AF and the frontal aslant tract (FAT) has been associated with single-word speech fluency in minimally verbal ASD individuals (Chenausky et al., 2017).

While DTI-derived metrics such as FA and MD are sensitive markers of WM integrity (Slater et al., 2019) and are the most frequently used to infer WM microstructure alterations, they are known to be non-specific, as they conflate several microstructural aspects, including axonal density and diameter, fiber coherence, membrane permeability and myelination (Beaulieu, 2002; Slater et al., 2019). Identifying which components of fiber microstructure, if any, are compromised in nvASD is essential for determining concrete neuropathological mechanisms. To this end, myelin water imaging (MWI), a multi-component T2 relaxometry technique, may achieve greater pathological specificity for individual tissue compartments (MacKay and Laule, 2016; Canales-Rodríguez et al., 2021a,2021b) and can thus complement DTI by discerning ambiguities inherent to single-modality metrics. Of particular interest is fiber myelination, a key regulator of neuronal network interactions, which develops dynamically in response to both intrinsic and experience-dependent factors (de Faria et al., 2021). Myelin water fraction (MWF) is a surrogate for myelin volume and shows a strong correlation with histology (MacKay and Laule, 2016), which is strongly linked to cognitive development (Deoni et al., 2012; Dean et al., 2015; Dai et al., 2019; Schneider et al., 2022) as well as age and gender-related microstructural changes (Canales-Rodríguez et al., 2021a). As such, MWF can provide invaluable insights into the neurobiological mechanisms underlying the absence of phrase speech and language development.

This study employed a multimodal neuroimaging approach to investigate WM microstructural abnormalities in nvASD. We examined a cohort of children with nvASD and age-matched typically developing control participants, assessing WM microstructure through a multiparametric framework. This included DTI metrics—FA, MD, and radial diffusivity (RD)—alongside MWI metrics, such as MWF and intra/extracellular water T2 relaxation time (T2IE).

The analysis focused on eight major language-related WM tracts: the superior longitudinal fasciculus (SLF-II and SLF-III), inferior longitudinal fasciculus (ILF), uncinate fasciculus (UF), middle longitudinal fasciculus (MLF), AF, IFOF, and FAT. The corticospinal tract (CST), a key motor pathway, was also examined as a reference, given a frequent lack of gross motor dysfunction in nvASD, and the possibility that WM alterations may or may not be specific to language tracts. First, we assessed the lateralization index for each tract, testing for within-group lateralization effects and inter-group differences. We then evaluated between-group differences in each WM microstructure metric. Finally, within the nvASD group, we explored associations between these microstructural metrics and speech comprehension and production levels.

## 2 Materials and methods

### 2.1 Sample

This study recruited 10 school-aged children and adolescents with nvASD, and 10 pairwise age-matched HC (Table 1). The nvASD group was recruited from several special schools and the Sant Joan de Déu Hospital in Barcelona. A subset of the nvASD sample (*N* = 8) was previously included in a broader study on cognitive profiling of this population (Slušná et al., 2021). It formed part of a preliminary previous DTI study targeting volume and FA-based hemispheric laterality of language tracts, using manual deterministic tractography (Olivé et al., 2022). The recruitment criteria were: (a) a parent or center-reported ASD diagnosis, (b) absence of phrase level-functional speech (defined based on a formal ASD classification) with a speech profile consisting of no words, single words, or fixed phrases as determined by parental, school, or center reports, and (c) evidence of absence of speech in other modalities (written and sign) or contexts (home, etc.). The speech status and diagnosis of the nvASD subjects were re-evaluated after recruitment throughout the study assessments with specific reference to Module 1 of The Autism Diagnostic Observation Schedule (ADOS) and The Autism Diagnostic Interview-Revised (ADI-R), except in two cases recruited from the hospital’s ASD unit. All nvASD subjects have idiopathic ASD except one subject with a 22q11 deletion syndrome.

**TABLE 1:** Demographics of the sample and cognitive vocalization profile of the nvASD group (note: age is stated as years; months).

**Subject**	nvASD									HC		
	Age	Sex	Handedness	VMA	NVIQ	ADOS score	Total of phonation instances	Total of articulation instances	Articulation-phonation ratio (%)	Age	Sex	Handedness*
1	8; 3	M	L	1; 3	85	15	461	190	−41.63	8; 11	M	R
2	9; 6	M	R	1; 0	53	13	72	118	24.21	9; 6	M	R
3	10; 11	M	R	1; 0	30	23	73	150	34.53	10; 1	M	R
4	11; 1	M	R	4; 3	79	18	92	139	20.35	11; 8	F	R
5	11; 1	M	R	1; 7	69	19	209	231	5	12; 1	M	R
6	11; 6	M	R-L*	N/A	N/A	N/A	N/A	N/A	N/A	12; 3	M	R
7	12; 3	F	R	1; 3	61	14	62	58	−3.33	12; 5	F	R
8	15; 1	M	R	1; 11	64	19	53	430	78.05	14; 6	M	R
9	16; 7	F	R	N/A	N/A	N/A	N/A	N/A	N/A	15; 11	F	R
10	17; 8	F	R	3; 11	69	22	8	113	86.78	17; 3	F	R

*Ambidextrous.

Table 1 summarizes the cognitive profile of the nvASD participants taken from a previous study (Slušná et al., 2021): non-verbal IQ was assessed with the Leiter International Performance Test-Revised (Roid et al., 2013), following recommendations for administration in low-functioning autism described in Tsatsanis et al. (2003), and verbal mental age was assessed with the Peabody Picture Vocabulary Test–III (PPVT-III) (Dunn and Dunn, 2007). Their speech-like vocalization profile was derived from the ADOS assessment after recruitment in a previous study (Sheets, 2022). The specific vocalization annotation scheme in that study was adapted from Buder et al. (2013), characterizing pre-speech vocalizations of typically developing infants. Volitional vocalizations of nvASD subjects were categorized as either phonated or articulated. Phonation (i.e., voicing) consists of primary, most rudimentary vocalizations produced by the rapid expansion and contraction of the vocal cords (vocants, squeals, and growls; see Buder et al., 2013). Increasingly similar to speech, articulated vocalizations are produced by the modulation of the vocal tract, resulting in a consonant-like sound or a closant-vocalic blend when syllabified (marginal syllables, canonical syllables, word approximations, or words).

MRI data from the HC group were obtained *in situ* using the same scanner parameters as those of the nvASD group. All HC children were born at term with no complications and no personal or family history of language delay or neurodevelopmental disorders [verified through adapted *TPBA2* Child and Family History Questionnaire, CFHQ (Linder, 2008)]. There was no statistically significant difference between the groups on age [mean difference = 0.02, *p* = 0.99, permutations = 100,00], sex [χ^2^(1,20) = 0.83, *p* = 0.36, permutations = 10,000] or handedness [χ^2^(1,20) = 1.05, *p* = 0.3, permutations = 10,000].

### 2.2 Diffusion MRI: data acquisition, preprocessing, and estimation

Diffusion MRI data were acquired for each participant using a 3T Ingenia CX scanner (Philips Medical Systems) located at the Hospital Sant Joan de Déu (Barcelona, Spain) with a standard 32-channel head coil and the following sequence parameters: Field-of-view = 230 mm × 230 mm; voxel-size = 2.05 mm × 2.05 mm; repetition time (TR) = 10.1 s; echo time (TE) = 102 ms; flip angle = 90°; number-of-slices = 64; slice-thickness = 2.1 mm; number of averages = 1; acceleration factor = 2; number of shells = 2; *b*-values = 625, and 1,250 s/mm^2^; number of diffusion gradient directions per *b*-value = 36; number of b0 (i.e., *b*-value = 0) images = 3, plus 1 b0 with reverse phase encoding to correct for spatial distortions.

All data preprocessing steps were performed using MRtrix3 (Tournier et al., 2019).^1^ Denoising was applied using the MP-PCA method (Veraart et al., 2016) (dwidenoise), followed by Gibbs ringing correction (Kellner et al., 2016) (mrdegibbs). Motion and eddy current distortions (Andersson and Sotiropoulos, 2016) were corrected using dwifslpreproc, while bias field inhomogeneities (Tustison et al., 2010) were addressed with dwibiascorrect. Brain extraction and masking (Jenkinson et al., 2005) were performed using dwi2mask. Finally, the diffusion tensor and derived parameter maps (FA, MD, and RD) were computed using dwi2tensor and tensor2metric. Raw data, as well as the outputs from each preprocessing and estimation step, were visually inspected as part of the quality control process. Given that propofol was administered as the intravenous anesthetic following the routine protocol at Sant Joan de Déu Hospital during the MRI acquisition, we did not observe significant motion artifacts, ensuring that all acquired subjects were included in the analyses.

### 2.3 Multi-echo T2 relaxometry: data acquisition, preprocessing, and estimation

Multi-echo T_2_ (MET_2_) data were acquired for all participants during the same scanning session described in the previous section by using a gradient and spin-echo sequence (Prasloski et al., 2012b) with the following parameters: Field-of-view = 240 mm × 230 mm; acquisition voxel-size = 3.5 mm × 3.5 mm, reconstructed voxel-size = 1.6 mm × 1.6 mm; number of echoes = 32; echo-time spacing (ΔTE) = 8.38 ms; repetition time (TR) = 8,620 ms; excitation pulse = 90°; refocusing pulses = 180°; number-of-slices = 40; slice-thickness = 3.5 mm; number of averages = 1; acceleration factor (SENSE) = 2; acquisition time = 8:37 min.

The intra-voxel T_2_ distribution of relaxation times was calculated by using regularized non-negative least squares (Laule et al., 2007; Mackay and Laule, 2012) with a regularization term to promote smooth solutions that better represent the distribution expected from tissue microstructure (Mackay et al., 1994; Whittall et al., 1997) as described in Canales-Rodríguez et al. (2021a) and Guo et al. (2013). The estimation was carried out using the open-source multi-component T_2_ reconstruction toolbox (Canales-Rodríguez et al., 2021b,2021c), available at https://github.com/ejcanalesr/multicomponent-T2-toolbox. The implementation is based on the extended phase graph (EPG) model (Prasloski et al., 2012a) using a T_2_ discrete grid from 10 to 2,000 ms (Prasloski et al., 2012b) with *p* = 60 T_2_ logarithmically spaced points.

From the estimated T_2_ distributions, the MWF was calculated as the area under the curve of the distribution for T_2_ times smaller than the myelin water cut-off T_2_ = 40 ms, normalized by the total area under the curve of the whole T_2_ distribution (Meyers et al., 2017). The T_2_ of intra- and extracellular water (T2IE) is computed as the geometric mean of the distribution in the T_2_ range 40–200 ms (Bjarnason, 2011).

### 2.4 Tract segmentation and estimation

TractSeg^2^ was used to perform tract segmentation (Wasserthal et al., 2018; Chandio et al., 2020). This tool leverages fiber Orientation Distribution Functions (fODFs) computed via constrained spherical deconvolution (CSD) (Dhollander et al., 2016), allowing for the identification of multiple fiber orientations within a voxel. This robust convolutional neural network-based approach directly segments tracts using the field of fODF peaks without using tractography and image registration. Although TractSeg can segment tracts directly in native space, we registered all the derived MRI maps to MNI space using a linear registration method (flirt) in FSL (Jenkinson et al., 2005). This step ensures consistent left/right orientation across all images, as recommended in the TractSeg documentation. We confirmed that this step produced results equivalent to those obtained with the “*–preprocess*” option in TractSeg, which automatically applies a rigid registration of the input image to MNI space, as well as to those obtained when processing was conducted entirely in native space.

From the segmented tract images, we calculated the mean value for each of the five MRI metrics used in this study (FA, MD, RD, MWF, and T2IE). Mean values were extracted from the nine tracts of interest, which included eight language-related tracts and one control tract. Values were computed separately for the left and right hemispheres. The spatial distribution of these segmented tracts for all participants is presented in Supplementary material (section 2) and was used as a quality control step.

### 2.5 Statistical analyses

All statistical analyses and visualizations were conducted using R (R Core Team, 2021) (version 4.4.3). Data preprocessing and visualization were performed using the tidyverse (Wickham et al., 2019) and ggplot2 (Wickham, 2016) packages. For each MRI-derived metric and tract, lateralization was quantified using a laterality index (*LI*), defined as (Benbadis et al., 1998):


$$L\,I=\frac{X_{L}-X_{R}}{X_{L}+X_{R}},$$

where *X*_*L*_ and *X*_*R*_ represent the mean values of the metric in the left and right hemispheres, respectively. With this definition, the index varies between +1 (strong left hemisphere dominance) and −1 (strong right hemisphere dominance). The statistical significance of lateralization (i.e., *LI* ≠ 0) for each tract was assessed using Wilcoxon signed-rank tests (Bauer, 1972) (wilcox.test function, base R), as the *LI* values may not always meet normality assumptions. Likewise, to test for group differences in *LI*, we used both Wilcoxon tests and Student’s *t*-tests. Since both analyses produced similar results, we only reported results from the Wilcoxon tests. To complement these analyses, 95% confidence intervals are reported, and effect sizes were estimated using the rank-biserial correlation (Kassambara, 2023) (rstatix:wilcox_effsize).

To evaluate inter-group differences, linear regression models were fitted for each metric (lm function, base R) with age included as a covariate: Y = β_0_ + β_1_ *Age* + β_2_ *Group* + ε, where Y represents the metric of interest, *Age* is a continuous covariate, and *Group* is a categorical variable. The model assumed a common slope for the age effect across groups. Regression coefficients, along with their 95% confidence intervals, were extracted using the confint function. Moreover, the percent of change of each metric was estimated for the nvASD group compared to the HC group.

Lastly, in the nvASD group, a correlation analysis was conducted to examine the relationship between each MRI-derived metric and several clinical variables (VMA, NVIQ, ADOS score, and articulation-phonation ratio; see Table 1). A linear model (lm function, base R) was fitted for each combination of tract and brain hemisphere, filtering the data to include only subjects with complete information on all clinical variables, resulting in 8 subjects from 10.

Results from the analyses were corrected for multiple comparisons using the false discovery rate (FDR) method at a significance level of *q* < 0.05 to control for the family-wise error rate (using the *p*.adjust function in R). This correction was applied independently to each MRI metric. We report both the raw *p* values and the FDR-corrected *p* values (denoted as p-FDR) to provide a comprehensive view of our findings.

### 2.6 Reanalysis using centroid-based FA estimation

Laterality index estimates have been shown to depend on the chosen preprocessing and analysis pipeline, particularly for the FA metric in the AF (Talozzi et al., 2018; Bain et al., 2019). As a complementary analysis aimed exclusively at assessing the robustness of our *LI* estimates, we repeated all FA-based *LI* analyses using an alternative quantification method provided by TractSeg. This method samples mean FA values along the centroid (i.e., central line) of each tract (Wasserthal et al., 2019).

Specifically, it employs probabilistic tracking based on estimated tract orientation maps (i.e., fODF peaks) to generate bundle-specific tractograms. All streamlines are first resampled into 100 segments, and the FA value is evaluated at each segment. The centroid of each tract is then computed, and the FA for each centroid segment is defined as the average FA across the nearest segments from surrounding streamlines. The final mean FA per tract is obtained by averaging the values from the 100 centroid segments (Wasserthal et al., 2020).

Importantly, this method and the approach described in section “2.4 Tract segmentation and estimation” (which involves averaging FA values over the entire tract mask) use the same preprocessed data. Therefore, any differences in *LI* between the two approaches reflect the influence of methodological choices on the estimation of tract-level FA. This reanalysis was conducted solely to assess the stability of our findings and to facilitate comparison with previous diffusion MRI studies using tractography-based *LI* estimates. It was not intended as a primary objective of this work.

### 2.7 Ethics

This study was approved by the responsible institutional review board (CEIC Fundació Sant Joan de Déu; PIC-99-17) in accordance with the 1964 Helsinki Declaration and its later amendments. Caregivers of all participants gave their written informed consent. The protocol included an intravenous-based anesthetic (propofol), which is the routine method used in the Sant Joan de Déu Hospital.

## 3 Results

Results for each MRI-derived metric are presented in separate subsections, tables, and figures. However, for clarity and ease of interpretation, all lateralization analysis results are combined into a single figure, with distinct panels for each metric. Only statistical findings that remain significant after FDR correction are reported.

### 3.1 Fractional anisotropy

Unless otherwise specified, all reported results in this section refer to the main FA-based *LI* analysis described in section “2.4 Tract segmentation and estimation.” Results from the centroid-based reanalysis are explicitly indicated when discussed.

**FIGURE 1:**
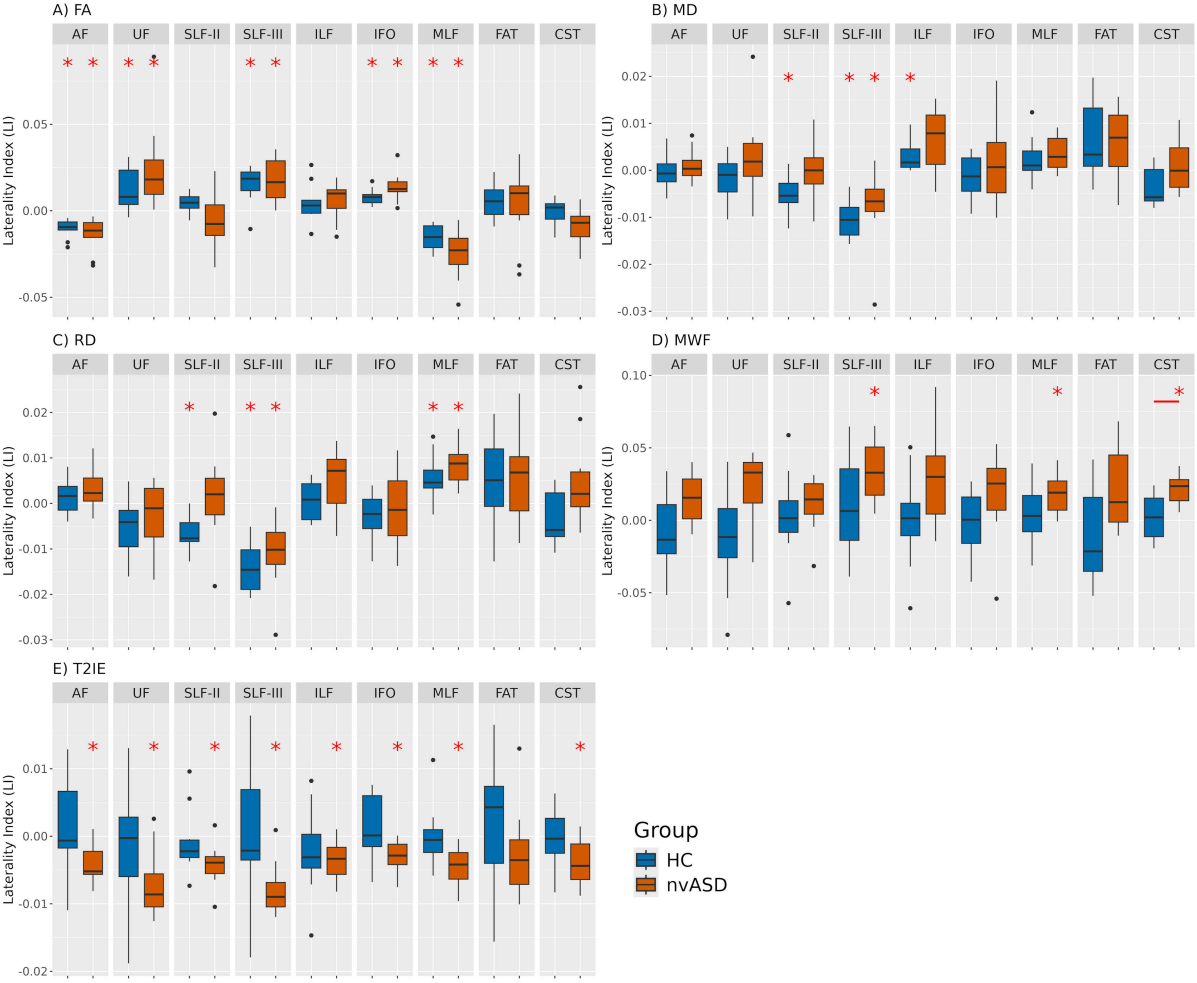
Laterality index (LI) values derived from five different MRI-based microstructural metrics for healthy controls (HC) and individuals with nvASD across nine WM tracts. Panels labeled **(A–E)** represent different metrics: FA, MD, RD, MWF, and T2IE, respectively. Significant LI values (LI 6 = 0) per tract, corrected for multiple comparisons within each MRI metric, are marked with a red asterisk. Significant inter-group differences are indicated by a red segment, with only one significant finding: the corticospinal tract (CST) for the MWF metric.

The lateralization results for the FA metric are presented in Figure 1A and Supplementary Tables 1, 2. Significant *LI* values were observed in five tracts across both groups. Specifically, the AF and MLF showed rightward lateralization (*LI* < 0), whereas the SLF-III, UF, and IFO were lateralized to the left hemisphere (*LI* > 0). Most *LI* values across subjects and tracts fell within the range of [−0.05, 0.05]. However, no significant between-group differences in *LI* were found between HC and the nvASD group in any tract.

In contrast, the centroid-based FA reanalysis yielded partially different patterns. No significant lateralization was observed in the AF, and although the SLF-III maintained positive *LI* values consistent with the main analysis, these were not statistically significant. The MLF exhibited leftward lateralization in the control group but was not significantly lateralized in the nvASD group. For further details, see Supplementary material (section 3).

**FIGURE 2:**
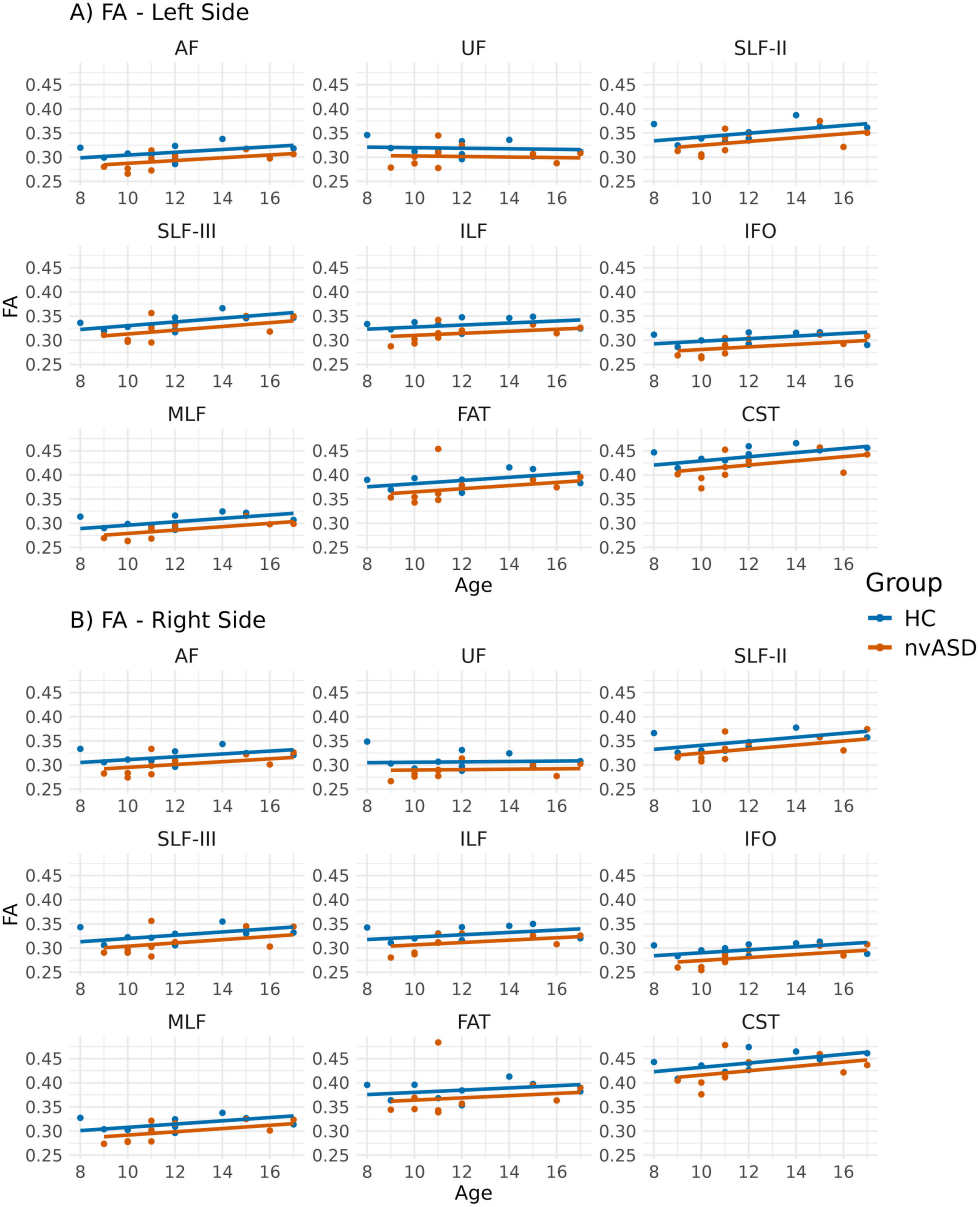
Scatter plots showing mean fractional anisotropy (FA) values per tract for all subjects. Data from tracts in the left and right hemispheres are shown in panels **(A,B)**, respectively. Two regression lines, one per group, are displayed to illustrate the relationship between FA and age, which is included as a covariate in the general linear model. Although age is not a variable of interest in this study, it is visualized here to support its role as a covariate in the analysis.

Inter-group FA differences, adjusted for age, are presented in Figure 2 and Table 2. The FA metric was significantly reduced in the nvASD group in the following tracts: left AF, left CST, left MLF, right IFO, right UF, and bilateral ILF, with a percentage reduction of approximately 6%–7% relative to the control group (see Table 2). In both groups, the highest FA values were observed in the CST and FAT, while the lowest were found in the IFO and UF (see Figure 2).

**TABLE 2:** Inter-group differences in fractional anisotropy (FA), adjusted for age.

Tract	Side	% of change	*p*-Value	*R* ^2^	CI-lower	CI-upper	p-FDR
AF	Left	−6.761	0.013	0.355	−0.033	−0.005	**0.033**
AF	Right	−5.991	0.039	0.267	−0.033	−0.001	0.084
CST	Left	−6.688	0.010	0.397	−0.045	−0.007	**0.033**
CST	Right	−4.911	0.084	0.248	−0.041	0.003	0.126
IFO	Left	−5.269	0.042	0.272	−0.028	−0.001	0.084
IFO	Right	−6.625	0.012	0.393	−0.030	−0.004	**0.033**
ILF	Left	−6.192	0.009	0.333	−0.033	−0.005	**0.033**
ILF	Right	−6.614	0.012	0.320	−0.035	−0.005	**0.033**
MLF	Left	−7.647	0.003	0.499	−0.032	−0.008	**0.033**
MLF	Right	−5.448	0.051	0.303	−0.030	0.000	0.092
SLF_III	Left	−4.548	0.100	0.292	−0.029	0.003	0.138
SLF_III	Right	−4.893	0.144	0.170	−0.033	0.005	0.173
SLF_II	Left	−5.960	0.064	0.264	−0.037	0.001	0.104
SLF_II	Right	−3.825	0.186	0.255	−0.029	0.006	0.210
UF	Left	−4.474	0.112	0.049	−0.033	0.004	0.144
UF	Right	−7.323	0.011	0.245	−0.039	−0.006	**0.033**
FAT	Left	−3.006	0.383	0.043	−0.035	0.014	0.405
FAT	Right	−2.419	0.582	−0.063	−0.041	0.024	0.582

The table includes tracts of interest, brain hemisphere, percentage change relative to the control group, *p* values and *R*^2^ from the regression analyses, confidence interval bounds (i.e., CI-lower and CI-upper), and FDR-adjusted *p* values (p-FDR) corrected for multiple comparisons. Statistically significant p-FDR values are highlighted in bold.

### 3.2 Mean diffusivity

The lateralization results for the MD metric are shown in Figure 1B, and detailed in Supplementary Tables 3, 4. Significant *LI* values were detected in both groups for a single tract, SLF-III, while additional lateralization effects were observed in SLF-II and ILF exclusively in the control group. Specifically, SLF-II and SLF-III exhibited rightward lateralization, whereas ILF was lateralized to the left. Across all subjects and tracts, the majority of *LI* values remained within the range of [−0.02, 0.02]. No significant group differences in *LI* were found for any tract when considering MD.

**FIGURE 3:**
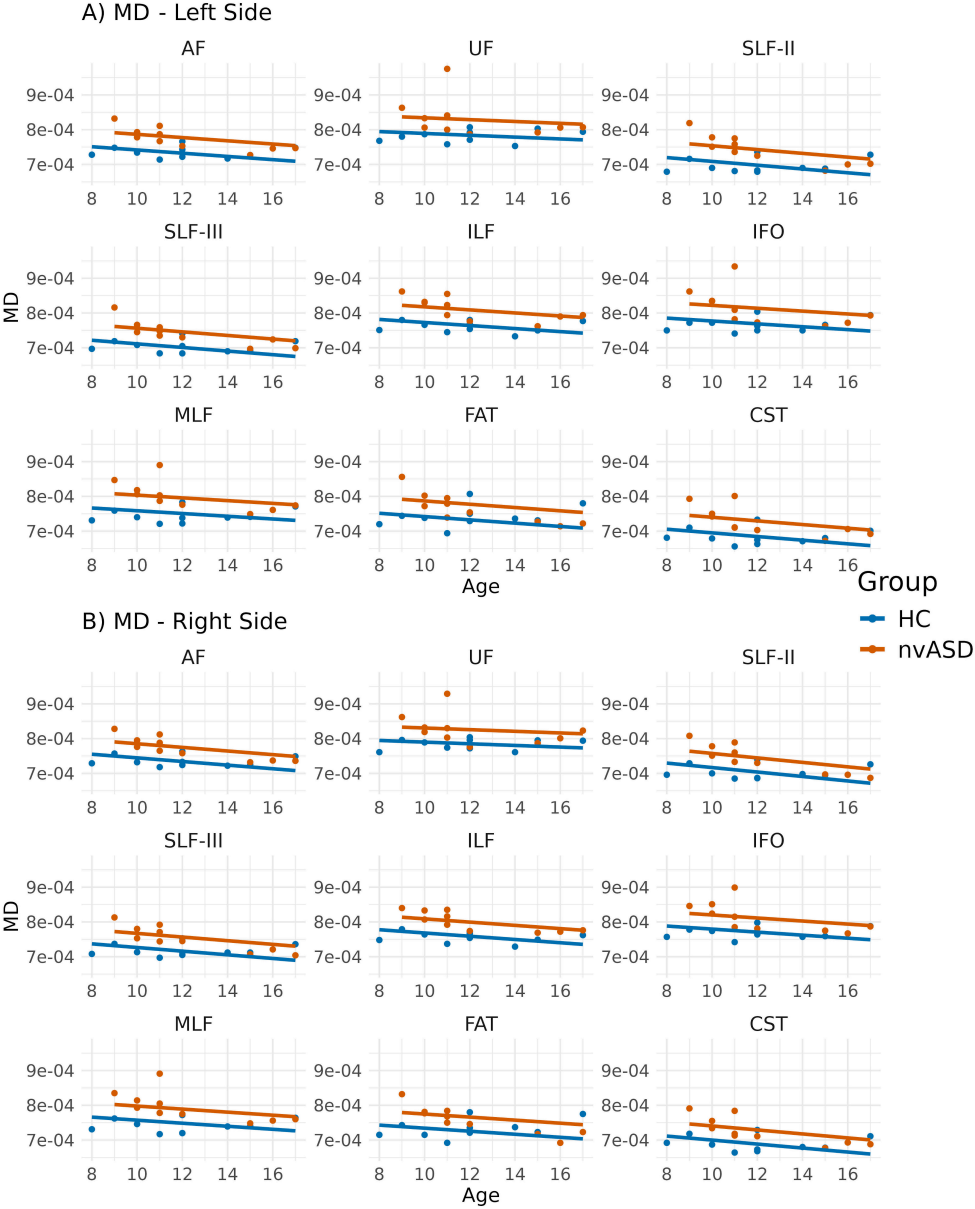
Scatter plots showing average mean diffusivity (MD) values per tract for all subjects. Data from tracts in the left and right hemispheres are shown in panels **(A,B)**, respectively. Two regression lines, one per group, are displayed to illustrate the relationship between MD and age, which is included as a covariate in the general linear model. Although age is not a variable of interest in this study, it is visualized here to support its role as a covariate in the analysis.

Age-adjusted inter-group differences in MD are presented in Figure 3 and Table 3. The nvASD group showed a significant increase in MD across all tracts except FAT, with differences ranging from approximately 4.5% to 7% relative to controls (see Table 3). In both groups, the lowest MD values were observed in CST, while UF exhibited the highest values (see Figure 3).

**TABLE 3:** Inter-group differences in mean diffusivity (MD), adjusted for age.

Tract	Side	% of change	*p*-Value	*R* ^2^	CI-lower	CI-upper	p-FDR
AF	Left	5.104	0.001	0.490	0.000	0.000	**0.004**
AF	Right	4.831	0.001	0.512	0.000	0.000	**0.004**
CST	Left	5.903	0.007	0.361	0.000	0.000	**0.010**
CST	Right	4.949	0.009	0.380	0.000	0.000	**0.013**
IFO	Left	6.154	0.011	0.280	0.000	0.000	**0.013**
IFO	Right	5.564	0.004	0.379	0.000	0.000	**0.008**
ILF	Left	6.427	<1e-4	0.575	0.000	0.000	**0.001**
ILF	Right	5.690	<1e-4	0.637	0.000	0.000	**0.001**
MLF	Left	7.239	0.001	0.450	0.000	0.000	**0.004**
MLF	Right	6.956	0.001	0.439	0.000	0.000	**0.004**
SLF_III	Left	5.098	0.002	0.460	0.000	0.000	**0.006**
SLF_III	Right	4.550	0.006	0.410	0.000	0.000	**0.010**
SLF_II	Left	6.122	0.003	0.416	0.000	0.000	**0.008**
SLF_II	Right	5.117	0.006	0.434	0.000	0.000	**0.010**
UF	Left	6.175	0.016	0.225	0.000	0.000	**0.018**
UF	Right	5.225	0.011	0.263	0.000	0.000	**0.013**
FAT	Left	3.010	0.167	0.097	−0.000	0.000	0.167
FAT	Right	3.090	0.121	0.126	−0.000	0.000	0.128

The table includes tracts of interest, brain hemisphere, percentage change relative to the control group, *p* values and *R*^2^ from the regression analyses, confidence interval bounds (i.e., CI-lower and CI-upper), and FDR-adjusted *p* values (p-FDR) corrected for multiple comparisons. Statistically significant p-FDR values are highlighted in bold.

### 3.3 Radial diffusivity

The lateralization findings for the RD metric are illustrated in Figure 1C, and detailed in Supplementary Tables 5, 6. Significant *LI* values were identified in both groups for the SLF-III and MLF tracts, while SLF-II exhibited significant lateralization only in controls. Specifically, SLF-II and SLF-III were right-lateralized, whereas MLF showed leftward lateralization. Across all tracts and participants, most *LI* values remained within the range of [−0.02, 0.02]. Consistent with previous DTI-based metrics, no significant group differences in *LI* were observed for RD in any tract.

**FIGURE 4:**
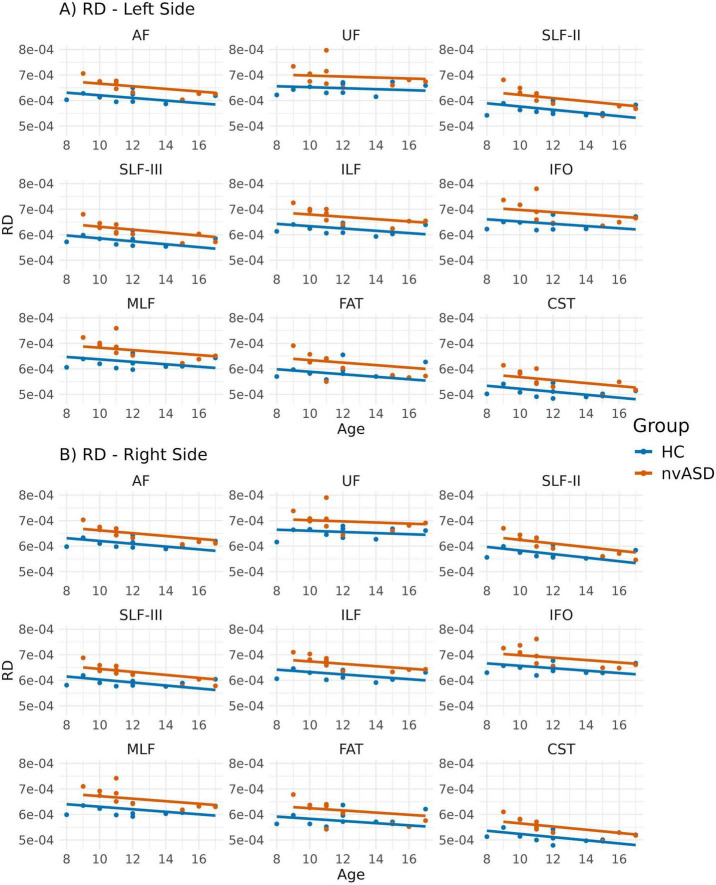
Scatter plots showing mean radial diffusivity (RD) values per tract for all subjects. Data from tracts in the left and right hemispheres are shown in panels **(A,B)**, respectively. Two regression lines, one per group, are displayed to illustrate the relationship between RD and age, which is included as a covariate in the general linear model. Although age is not a variable of interest in this study, it is visualized here to support its role as a covariate in the analysis.

Group comparisons for RD, adjusted for age, are summarized in Figure 4 and Table 4. The nvASD group displayed significantly higher RD values across all tracts except FAT, with increases ranging from approximately 5.5% to 8.5% relative to controls (see Table 4). In both groups, CST exhibited the lowest RD values, while the highest values were found in UF and IFO (see Figure 4).

**TABLE 4:** Inter-group differences in radial diffusivity (RD), adjusted for age.

Tract	Side	% of change	*p*-Value	*R* ^2^	CI-lower	CI-upper	p-FDR
AF	Left	6.331	0.001	0.540	0.000	0.000	**0.002**
AF	Right	5.948	0.001	0.556	0.000	0.000	**0.002**
CST	Left	8.419	0.001	0.508	0.000	0.000	**0.002**
CST	Right	6.738	0.002	0.519	0.000	0.000	**0.004**
IFO	Left	7.117	0.006	0.333	0.000	0.000	**0.007**
IFO	Right	6.777	0.002	0.421	0.000	0.000	**0.004**
ILF	Left	7.923	<1e-4	0.601	0.000	0.000	**0.001**
ILF	Right	7.088	<1e-4	0.611	0.000	0.000	**0.001**
MLF	Left	8.664	0.001	0.500	0.000	0.000	**0.002**
MLF	Right	8.098	0.001	0.513	0.000	0.000	**0.002**
SLF_III	Left	5.950	0.002	0.488	0.000	0.000	**0.004**
SLF_III	Right	5.401	0.004	0.470	0.000	0.000	**0.005**
SLF_II	Left	7.348	0.002	0.475	0.000	0.000	**0.004**
SLF_II	Right	5.803	0.004	0.496	0.000	0.000	**0.006**
UF	Left	7.449	0.005	0.306	0.000	0.000	**0.007**
UF	Right	6.951	0.005	0.313	0.000	0.000	**0.007**
FAT	Left	3.468	0.199	0.088	−0.000	0.000	0.202
FAT	Right	3.427	0.202	0.069	−0.000	0.000	0.202

The table includes tracts of interest, brain hemisphere, percentage change relative to the control group, *p* values and *R*^2^ from the regression analyses, confidence interval bounds (i.e., CI-lower and CI-upper), and FDR-adjusted *p* values (p-FDR) corrected for multiple comparisons. Statistically significant p-FDR values are highlighted in bold.

### 3.4 Myelin water fraction

The lateralization analysis for the MWF metric is shown in Figure 1D and Supplementary Tables 7, 8. Significant *LI* values were identified exclusively in the nvASD group for three tracts: SLF-III, MLF, and CST, all exhibiting leftward lateralization. No significant lateralization effects were detected in the control group. Across all subjects and tracts, most LI values remained within the range of [−0.07, 0.07]. A significant group difference in *LI* was found in the CST.

**FIGURE 5:**
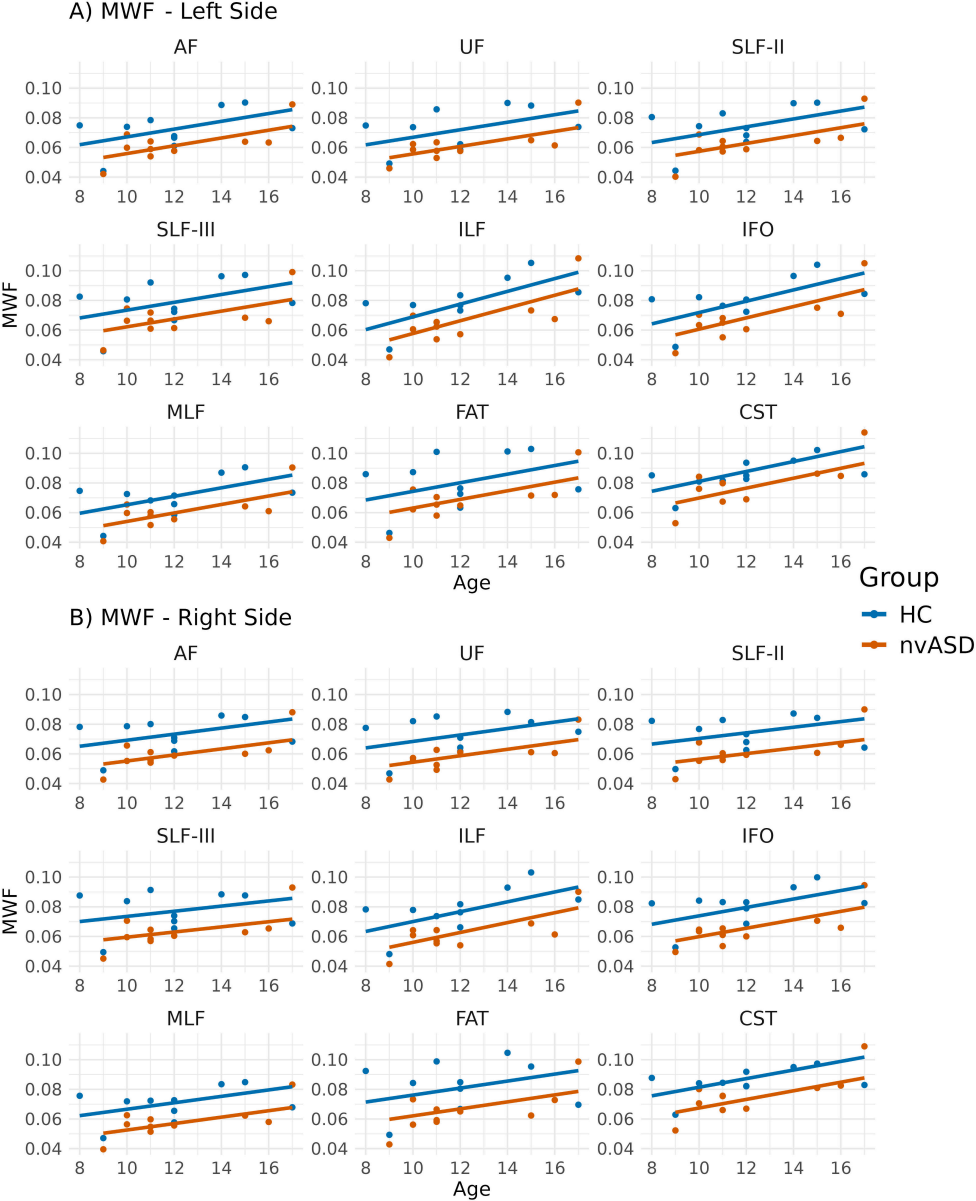
Scatter plots showing mean myelin water fraction (MWF) values per tract for all subjects. Data from tracts in the left and right hemispheres are shown in panels **(A,B)**, respectively. Two regression lines, one per group, are displayed to illustrate the relationship between MWF and age, which is included as a covariate in the general linear model. Although age is not a variable of interest in this study, it is visualized here to support its role as a covariate in the analysis.

Age-adjusted group differences in MWF are summarized in Figure 5 and Table 5. Compared to controls, the nvASD group showed significantly lower MWF values in several tracts, including bilateral IFO and ILF, as well as the right AF, MLF, UF, and FAT, with reductions ranging from 25% to 50% (see Table 5). In both groups, CST displayed the highest MWF values, while MLF exhibited the lowest (see Figure 5).

**TABLE 5:** Inter-group differences in myelin water fraction (MWF), adjusted for age.

Tract	Side	% of change	*p*-Value	*R* ^2^	CI-lower	CI-upper	p-FDR
AF	Left	−25.286	0.048	0.353	−0.020	−0.000	0.057
AF	Right	−26.288	0.014	0.355	−0.022	−0.003	**0.041**
CST	Left	−14.718	0.155	0.433	−0.016	0.003	0.155
CST	Right	−19.138	0.041	0.427	−0.019	−0.000	0.056
IFO	Left	−39.224	0.015	0.519	−0.024	−0.003	**0.041**
IFO	Right	−35.574	0.002	0.539	−0.026	−0.007	**0.016**
ILF	Left	−50.510	0.022	0.531	−0.025	−0.002	**0.049**
ILF	Right	−45.322	0.002	0.569	−0.027	−0.007	**0.016**
MLF	Left	−28.915	0.039	0.399	−0.020	−0.001	0.056
MLF	Right	−27.200	0.013	0.387	−0.021	−0.003	**0.041**
SLF_III	Left	−23.495	0.071	0.276	−0.023	0.001	0.075
SLF_III	Right	−23.929	0.026	0.250	−0.025	−0.002	0.052
SLF_II	Left	−26.797	0.042	0.340	−0.022	−0.000	0.056
SLF_II	Right	−23.474	0.029	0.277	−0.022	−0.001	0.052
UF	Left	−25.869	0.044	0.338	−0.021	−0.000	0.056
UF	Right	−32.249	0.004	0.431	−0.025	−0.005	**0.026**
FAT	Left	−29.243	0.058	0.273	−0.028	0.001	0.065
FAT	Right	−32.578	0.016	0.306	−0.032	−0.004	**0.041**

The table includes tracts of interest, brain hemisphere, percentage change relative to the control group, *p* values and *R*^2^ from the regression analyses, confidence interval bounds (i.e., CI-lower and CI-upper), and FDR-adjusted *p* values (p-FDR) corrected for multiple comparisons. Statistically significant p-FDR values are highlighted in bold.

### 3.5 T_2_ relaxation time of intra- and extra-axonal water

The lateralization analysis for the T2IE metric is shown in Figure 1E and Supplementary Tables 9, 10. Significant *LI* values were identified exclusively in the nvASD group for all the tracts except FAT, all of which exhibited rightward lateralization. No significant lateralization effects were detected in the control group. Across all subjects and tracts, most LI values are within the range of [−0.01, 0.01]. No significant group differences in *LI* were observed for T2IE in any tract.

**FIGURE 6:**
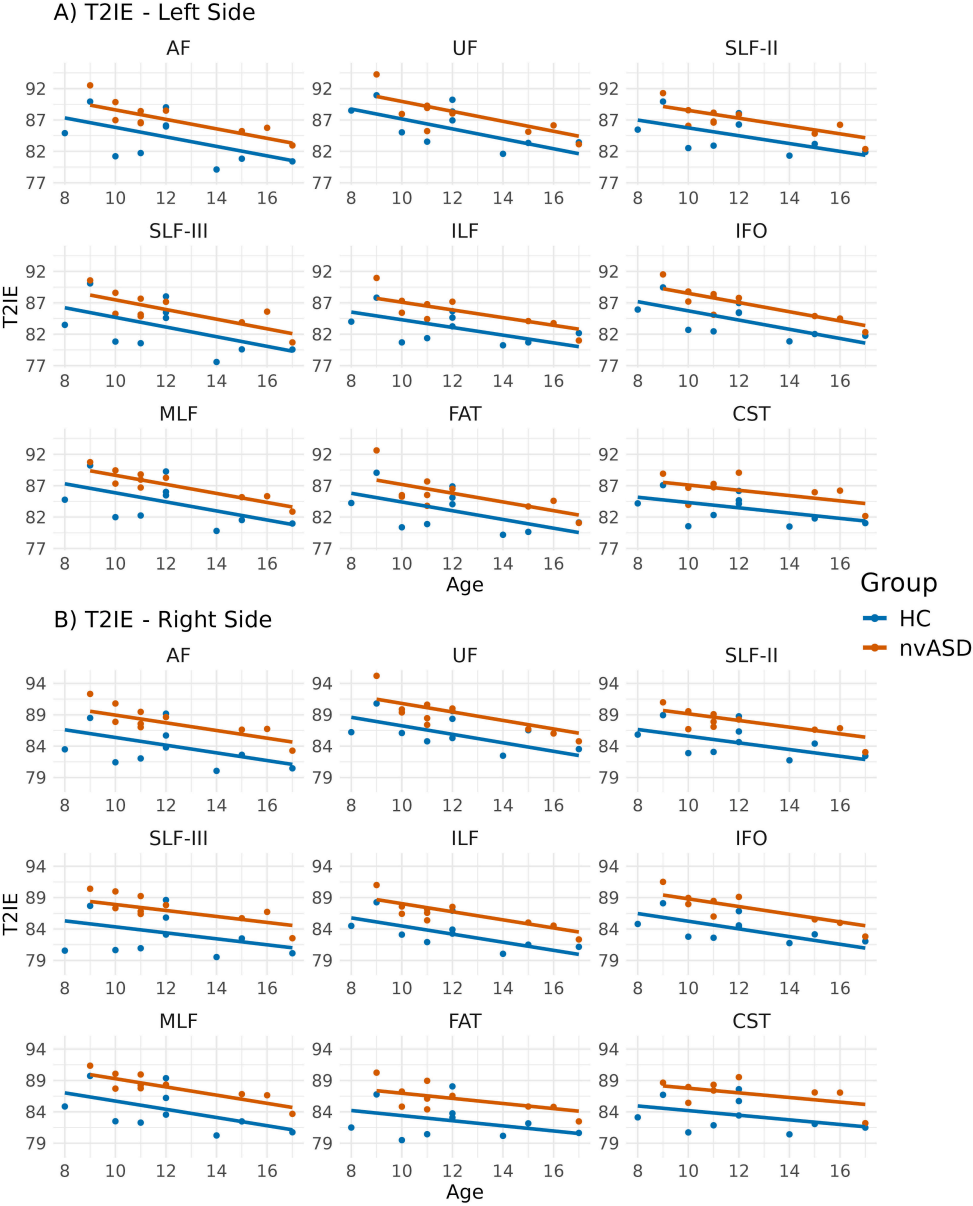
Scatter plots showing mean T2 relaxation times of the intra- and extra-axonal water (T2IE) per tract for all subjects. Data from tracts in the left and right hemispheres are shown in panels **(A,B)**, respectively. Two regression lines, one per group, are displayed to illustrate the relationship between T2IE and age, which is included as a covariate in the general linear model. Although age is not a variable of interest in this study, it is visualized here to support its role as a covariate in the analysis.

Age-adjusted group differences in T2IE are presented in Figure 6 and Table 6. Compared to controls, the nvASD group showed significantly higher T2IE values in all the tracts except left UF. The T2IE increases ranged from 3% to 5% (see Table 6). In both groups, ILF, FAT, and MLF displayed the lowest T2IE values, while UF exhibited the highest (see Figure 6).

**TABLE 6:** Inter-group differences in T2 relaxation times of the intra- and extra-axonal water (T2IE), adjusted for age.

Tract	Side	% of change	*p*-value	*R* ^2^	CI-lower	CI-upper	p-FDR
AF	Left	3.823	0.007	0.497	1.127	5.989	**0.009**
AF	Right	4.858	0.001	0.564	2.220	6.636	**0.004**
CST	Left	3.696	0.002	0.485	1.428	5.101	**0.004**
CST	Right	4.403	0.001	0.489	1.862	5.867	**0.004**
IFO	Left	2.900	0.005	0.606	0.958	4.442	**0.008**
IFO	Right	3.712	0.000	0.664	1.858	4.929	**0.004**
ILF	Left	3.135	0.006	0.511	0.934	4.736	**0.009**
ILF	Right	3.321	0.001	0.624	1.407	4.647	**0.004**
MLF	Left	3.424	0.007	0.519	1.016	5.346	**0.009**
MLF	Right	4.331	0.001	0.577	1.923	6.054	**0.004**
SLF_III	Left	3.380	0.024	0.425	0.474	5.762	**0.028**
SLF_III	Right	5.000	0.001	0.472	2.000	6.875	**0.004**
SLF_II	Left	2.396	0.028	0.451	0.267	4.151	**0.032**
SLF_II	Right	3.075	0.004	0.497	1.016	4.594	**0.008**
UF	Left	1.638	0.154	0.443	−0.648	3.779	0.154
UF	Right	2.761	0.014	0.493	0.600	4.613	**0.018**
FAT	Left	2.956	0.034	0.397	0.233	5.171	**0.036**
FAT	Right	4.017	0.005	0.388	1.236	5.795	**0.008**

The table includes tracts of interest, brain hemisphere, percentage change relative to the control group, *p* values and *R*^2^ from the regression analyses, confidence interval bounds (i.e., CI-lower and CI-upper), and FDR-adjusted *p* values (p-FDR) corrected for multiple comparisons. Statistically significant p-FDR values are highlighted in bold.

### 3.6 Association between MRI-derived metrics and clinical variables

The correlation analyses conducted in the nvASD group to explore potential relationships between each MRI-derived metric and key clinical variables, including VMA, NVIQ, ADOS score, and articulation-phonation ratio (see Table 1), did not reveal any significant results.

## 4 Discussion

This study investigated the neurobiological mechanisms associated with the lack of phrase speech development in nvASD. We tackled differences in the microstructure of language-related WM pathways, specifically, the dorsal and ventral routes linked to speech production and comprehension. In addition to conventional DTI parameters estimated from diffusion MRI data, the study utilized a MWI technique with greater specificity to individual tissue compartments, particularly the MWF as an index of myelin content. The cortico-spinal tract involved in motor control was also examined to discern characteristics specific to language-related circuity.

Our findings reveal that while DTI metrics, FA, MD, and RD exhibit similar lateralization patterns in both typically developing children (control group) and the nvASD group, MWF and T2IE metrics demonstrate significant lateralization exclusively within the patient group. This observation suggests that these neuroimaging metrics capture complementary aspects of brain tissue microstructure. Notably, inter-group comparisons of the lateralization index across various tracts and metrics revealed no significant differences, except for the CST tract when assessed using the MWF metric.

Interpreting these results in the context of existing literature is challenging due to the limited number of studies focusing on lateralization indices in nvASD. For instance, a study by Wan et al. (2012) reported reversed lateralization in the volume of the AF in five nvASD cases. In contrast, a more recent study with a larger sample size found no significant differences in either the AF’s volume or its left-hemispheric lateralization in terms of FA (Olivé et al., 2022). On the other hand, a functional MRI study found reduced lateralization of multiple functional brain networks in autistic males (Peterson et al., 2024). However, it is important to note that functional metrics and volumetric measures that reflect macroscopic properties may not directly correspond with microstructural properties such as FA or other metrics employed in our study. Our findings contribute to the growing body of evidence indicating that lateralization patterns in nvASD are complex and may vary depending on the specific neuroimaging metric and brain region assessed.

Our FA-based lateralization results in HC showed both similarities and differences compared to previous studies. For instance, we observed rightward lateralization of FA in the AF, whereas Thiebaut de Schotten et al. (2011) did not report significant FA-based lateralization in a sample of 40 healthy adults using DTI-based deterministic tractography. However, although their group-level findings did not survive correction for multiple comparisons, they did note that individual FA values tended to be higher in the left than in the right AF. Interestingly, among the three segments of the AF, the anterior segment showed rightward asymmetry, while the long segment showed a clear (though statistically non-significant) trend toward leftward FA asymmetry (Thiebaut de Schotten et al., 2011). Given that different AF segments may exhibit distinct lateralization patterns, inter-study differences could be partially attributed to variations in how the AF is defined and segmented across methods.

Our findings for the IFO are consistent with Thiebaut de Schotten et al. (2011), as both studies found leftward FA lateralization. Additionally, Angstmann et al. (2016) reported significant leftward FA lateralization in the CST in a group of 52 typically developing adolescents (aged 11–16), using a CST mask derived from the TBSS approach (Smith et al., 2006). In contrast, we did not observe significant FA lateralization in the CST. Our result aligns more closely with that of Thiebaut de Schotten et al. (2011), who found no significant FA-based lateralization in this tract. It is worth noting that in their study, the reported leftward asymmetry was related to CST volume, not FA. For the SLF-III, previous work using CSD-based tractography in both adolescents and adults reported rightward FA lateralization (Amemiya et al., 2021), whereas we observed leftward lateralization in this tract.

To further investigate the potential influence of methodological choices, we repeated all FA-based *LI* analyses using an alternative quantification method available in TractSeg, which samples mean FA values along the tract’s centroid rather than across the full tract mask. In this reanalysis, the AF no longer showed significant lateralization, with a non-significant trend toward leftward *LI*, more in line with previous literature (Thiebaut de Schotten et al., 2011). In the SLF-III, *LI* values remained positive but lost statistical significance. Interestingly, the MLF, which initially showed rightward lateralization in both groups, appeared left-lateralized in controls and non-significant in the nvASD group after reanalysis.

These findings reinforce the idea that lateralization indices are sensitive to the specific method or pipeline used to estimate tract-based metrics. The discrepancies we observed are consistent with prior studies (Talozzi et al., 2018; Bain et al., 2019). While deterministic tractography based on single-fiber DTI models may yield more consistent lateralization patterns for some tracts, such as the AF, probabilistic tracking based on multi-fiber models may be better suited for others. An additional confounding factor is the common assumption that all streamlines within a tract follow the same lateralization pattern, which may not hold for all tracts particularly in the case of the AF (Thiebaut de Schotten et al., 2011). As a result, different tract segmentation approaches may capture different portions of a bundle, leading to variability in laterality estimates. Currently, there is no universally accepted gold standard, and different tracts may require distinct quantification strategies depending on their anatomical and microstructural complexity. In this study, we employed TractSeg, as a recent evaluation comparing several state-of-the-art bundle segmentation techniques showed that it produced more accurate results (i.e., higher Dice scores) across 72 reference bundles (Wasserthal et al., 2018).

The analyses comparing MRI-derived metrics between groups revealed microstructural abnormalities in the nvASD group. Notably, FA—the most widely used DTI metric for studying WM—was not the most sensitive for detecting inter-group differences. Instead, MD and RD exhibited significant differences across nearly all tracts, with the exception of the FAT bilaterally (see Tables 2–4). Interestingly, the FAT, a tract that connects the superior and inferior frontal gyri and has been implicated in various motor speech disorders including apraxia of speech (Zhong et al., 2022), does not appear to be affected in the nvASD group when using DTI measures. These findings, based solely on DTI metrics, could be attributed to various factors, including reduced axonal packing density (i.e., increased extra-axonal volume fraction), a decrease in outer axon diameters potentially linked to lower myelin volume, increased local axonal orientation dispersion, or a combination of these factors. Our results align with previous ASD studies (Walker et al., 2012; Weber et al., 2022) reporting reduced FA alongside increased MD and RD (Li et al., 2022).

Both MWF and T2IE metrics also revealed significant inter-group differences. MWF exhibited the largest percentage change relative to controls (25%–50%), suggesting a marked reduction in myelin volume in the nvASD group. However, despite this pronounced decrease, only a subset of tracts showed statistically significant differences (see Table 5). This may be due to the higher uncertainty of MWF estimates derived from regularized non-negative least squares methods (Canales-Rodríguez et al., 2021b,2021c), given the ill-posed nature of the inversion process. Conversely, although T2IE displayed smaller percentage changes (3%–5%), it achieved statistical significance in nearly all tracts except the left UF. The greater sensitivity of T2IE compared to MWF could be attributed to its higher estimation robustness (Fischi-Gomez et al., 2022).

Considering all our findings together, the results indicate a significant reduction in myelin volume, leading to a less densely packed extra-axonal space and smaller outer axon radii in the nvASD group. This hypothesis is supported by multiple observations: a decreased MWF, which is associated with lower myelin volume; an increased T2IE, which is inversely proportional to the surface-to-volume ratio of cells (Barakovic et al., 2023; Canales-Rodríguez et al., 2023, 2024)—suggesting that a reduced myelin surface area per unit of volume leads to higher T2IE values; and an elevated RD, indicative of less restricted molecular diffusion in the extra-axonal space. These changes also contribute to the observed MD increase and FA reduction in the nvASD group.

Notably, our findings align with recent evidence suggesting that myelin disruptions are critical in the pathophysiology of ASD. For instance, mutations in the CHD8 gene, frequently associated with ASD (Bernier et al., 2014), regulate myelination processes (Zhao et al., 2018). Molecular genetic studies have also revealed aberrations in myelin-related genes concerning expression levels and epigenetic regulation (Richetto et al., 2017). Post-mortem histological studies demonstrate abnormal morphology in myelin and axons in ASD (Zikopoulos and Barbas, 2010), specifically showing area-specific changes below the anterior cingulate cortex (ACC) characterized by a decrease in the largest axons that communicate over long distances, along with an excessive number of thin axons linking neighboring areas. Additionally, in the orbitofrontal cortex, axons exhibited reduced myelin thickness (Zikopoulos and Barbas, 2010). A recent review highlighted the crucial role of oligodendrocytes and myelin in ASD’s pathophysiology, reporting reductions in myelin sheath thickness, WM density, and long-range connections, alongside an increase in short-range connections (Galvez-Contreras et al., 2020). Furthermore, a recent neuroimaging study found widespread increases in extracellular water in the cortex of individuals with ASD, accompanied by decreases in g-ratio and conduction velocity throughout the cortex, subcortex, and WM (Newman et al., 2024).

The formation and maturation of myelin sheaths is a prolonged and dynamic process that extends over several decades, occurring in a non-linear manner with distinct waves of rapid and synchronized myelination events (de Faria et al., 2021). The first of these critical periods begins at birth and continues throughout the early years of life, closely paralleling the rapid development of cognitive and linguistic abilities (O’Muircheartaigh et al., 2014; Deoni et al., 2016). Current myelination models propose that both intrinsic and adaptive factors contribute to this process: an oligodendrocyte-intrinsic program establishes an initial pattern of myelination, while external signals, such as neuronal activity and environmental experiences, refine and modify myelin structure over time (Bechler et al., 2018). From this perspective, a reduction in extrinsic signaling, such as decreased axonal activity, could result in the formation of myelin sheaths that are either smaller in size or reduced in number. Our findings align with this framework, suggesting a substantial decrease in myelin content in nvASD, potentially influenced by early disruptions in axonal signaling. Given that myelination plays a crucial role in efficient neural communication, an alteration in its early developmental trajectory could have long-lasting effects on cognitive and motor functions.

The observed microstructural differences in language-related WM tracts in nvASD may reflect disruptions in early myelination, mirroring the delayed or impaired language acquisition characteristic of this condition. However, despite this potential link, our correlation analyses did not reveal significant associations between MRI-derived metrics and key clinical variables (VMA, NVIQ, ADOS score, and articulation-phonation ratio). This suggests that these clinical measures may not fully capture the variability in myelination-related changes within language-related WM tracts or that additional neurobiological factors contribute to these alterations in nvASD. Another potential explanation could be the relatively small sample size of our study. For example, in contrast to our study, Chenausky et al. (2017) predicted treatment-based change in speech accuracy in minimally verbal ASD individuals from the FA values of the FAT and AF tracts. Thus, future studies using these metrics should be conducted with larger sample sizes to obtain more conclusive results.

Notably, the inter-group differences in microstructure metrics were not restricted to language-related tracts but also involved the CST. This finding indicates that the observed microstructural anomalies in nvASD extend beyond the neural pathways specifically associated with language. Our results may align with previous studies reporting motor deficits in autistic individuals (Lim et al., 2021; Reindal et al., 2022; Kangarani-Farahani et al., 2024; Maffei et al., 2024). Additionally, a preliminary analysis (not included in this study) of the optic radiation revealed similar alterations to those found in the CST, further reinforcing the notion of widespread WM abnormalities in nvASD. This preliminary result might agree with previous studies reporting alterations in the visual system (Seymour et al., 2019; Chung and Son, 2020; Zhou et al., 2023). These findings suggest that atypical myelination patterns in nvASD may not be confined to tracts underlying linguistic functions but could instead reflect a more generalized alteration in WM development, potentially impacting multiple cognitive and other domains.

In summary, the study revealed widespread microstructural anomalies in language- and motor-related pathways in individuals with nvASD. These anomalies were detected through various MRI metrics, each capturing specific aspects of tissue microstructure. All the observed changes may be explained by a reduction in myelin volume or the number of myelinated axons in the nvASD group. Our findings suggest that research on nvASD should extend beyond examining isolated brain structures and instead prioritize comprehensive profiling of the entire brain using multiple quantitative metrics. Our results also indicated that certain imaging metrics could be more sensitive than others to detect microstructural changes.

## 5 Limitations

The findings of this study should be interpreted with caution due to several limitations. The sample size was limited, a common challenge in clinical research involving severely impaired and uncooperative populations. The cut-off was based on prior DTI studies in this population, which included ten or fewer participants and still reported significant findings. Although the control group was well-matched for chronological age, sex and handedness were only loosely controlled, ensuring no significant group differences but leaving some variability. Additionally, age-related changes were not assessed due to the small sample size and lack of longitudinal data.

To minimize variability in tract segmentation, we employed a state-of-the-art method (TractSeg) that has demonstrated superior accuracy compared to alternative approaches (Wasserthal et al., 2018). Nevertheless, different segmentation techniques can yield variations in tract reconstructions (Schilling et al., 2021), which may influence the results and their interpretation (Bain et al., 2019). To assess the impact of such methodological choices, we repeated all FA-based *LI* analyses using an alternative quantification strategy within TractSeg. This reanalysis led to partially divergent lateralization patterns. These findings highlight that lateralization indices are sensitive to the specific methods and pipelines used to estimate tract-based metrics. They also underscore the importance of interpreting laterality findings in diffusion MRI with caution and of explicitly accounting for methodological variability when comparing results across pipelines or studies. Consequently, our results should be interpreted cautiously until replicated in independent samples and using alternative methodologies. Future efforts to standardize tract segmentation protocols and lateralization quantification strategies will be essential to improve reproducibility and comparability across studies.

While tract selection was theoretically motivated, future research should explore whether the distinct anomalies observed in language-related tracts and CST also extend to other fibers with similar maturational trajectories, given the broad cognitive difficulties characteristic of the nvASD population (Slušná et al., 2021).

Regarding the employed MRI techniques, DTI and MWI are powerful techniques but have notable limitations. DTI assumes Gaussian diffusion, which oversimplifies complex microstructural environments, leading to biases in regions with crossing fibers or highly restricted diffusion. Partial volume effects, susceptibility artifacts, and noise can further distort tensor-derived metrics like FA, MD, and RD. Similarly, MWI relies on multi-exponential T2 relaxometry, which is sensitive to the chosen inversion model, estimation method, and regularization constraints. Factors like iron content, fiber orientation, and B1 inhomogeneities can confound interpretations in both techniques. Additionally, linking derived metrics to specific biological properties requires caution, as multiple microstructural features may influence the same parameter, complicating direct biological inferences.

Finally, it is important to note that this study only compared typically developing HC and individuals with nvASD. Future studies could benefit from including a third group of ASD individuals without the speech deficits characteristic of nvASD. This would help to delineate which differences are specific to the nvASD group in relation to the broader ASD population.

## Data Availability

The raw MRI data used in this study involve children with autism and cannot be shared publicly in order to protect participant privacy, in accordance with ethical guidelines and institutional regulations. Requests to access these datasets should be directed to the corresponding author.

## Footnotes

1
https://www.mrtrix.org/

2
https://github.com/MIC-DKFZ/TractSeg.git
