# Supplementary material for: White matter microstructure of language pathways in non-verbal autism: insights from diffusion tensor imaging and myelin water imaging
Source: Front Hum Neurosci. 2025 Aug 22;19:1551868. doi: 10.3389/fnhum.2025.1551868 (PMC12411538; doi:10.3389/fnhum.2025.1551868)
Supplement: Supplementary file 1 [file Data_Sheet_1.pdf]

Supplementary Material:

White Matter Microstructure of Language Pathways in Nonverbal Autism: Insights from Diffusion Tensor Imaging and Myelin Water Imaging

1. Supplementary Tables

The following subsections present the lateralization index (LI) results for each MRI metric. The between-group analyses correspond to the inter-group comparisons per tract, while the within-group analyses refer to the lateralization results for each group separately.

1.1 Fractional Anisotropy (FA)

Table S1: Between-groups LI results. Statistically significant p-FDR values are highlighted in bold.

Tract	p-value	effect-size	CI-lower	CI-upper	p-FDR
AF	0.529	0.152	-0.004	0.009	0.680
UF	0.123	0.355	-0.024	0.008	0.258
SLF_II	0.089	0.389	-0.001	0.020	0.258
SLF_III	0.853	0.051	-0.014	0.013	0.853
ILF	0.481	0.169	-0.013	0.011	0.680
IFO	0.123	0.355	-0.011	0.001	0.258
MLF	0.143	0.338	-0.003	0.021	0.258
FAT	0.631	0.118	-0.017	0.015	0.709
CST	0.089	0.389	-0.002	0.018	0.258

Table S2: Within-groups LI results. Statistically significant p-FDR values are highlighted in bold.

Tract	Group	p-value	effect-size	CI-lower	CI-upper	p-FDR
AF	HC	0.002	0.000	-0.014	-0.007	**0.004**
AF	nvASD	0.002	0.000	-0.021	-0.007	**0.004**
UF	HC	0.014	16.128	0.002	0.022	**0.025**
UF	nvASD	0.002	17.393	0.010	0.049	**0.004**
SLF_II	HC	0.037	15.179	0.000	0.009	0.061
SLF_II	nvASD	0.275	5.060	-0.014	0.006	0.310
SLF_III	HC	0.010	16.444	0.008	0.023	**0.020**
SLF_III	nvASD	0.002	17.393	0.008	0.029	**0.004**
ILF	HC	0.193	12.965	-0.002	0.012	0.249
ILF	nvASD	0.232	12.649	-0.002	0.015	0.279
IFO	HC	0.002	17.393	0.005	0.011	**0.004**
IFO	nvASD	0.002	17.393	0.008	0.019	**0.004**
MLF	HC	0.002	0.000	-0.022	-0.010	**0.004**
MLF	nvASD	0.002	0.000	-0.036	-0.013	**0.004**
FAT	HC	0.193	12.965	-0.003	0.013	0.249
FAT	nvASD	0.432	11.384	-0.012	0.021	0.457
CST	HC	0.922	9.171	-0.006	0.005	0.922
CST	nvASD	0.064	2.846	-0.016	0.000	0.097

1.2 Mean Diffusivity (MD): Lateralization results

Table S3: Between-groups LI results. Statistically significant p-FDR values are highlighted in bold.

Tract	p-value	effect-size	CI-lower	CI-upper	p-FDR
AF	0.481	0.169	-0.005	0.002	0.619
UF	0.190	0.304	-0.011	0.002	0.428
SLF_II	0.054	0.440	-0.010	0.000	0.242
SLF_III	0.123	0.355	-0.009	0.001	0.369
ILF	0.280	0.254	-0.011	0.002	0.504
IFO	0.677	0.101	-0.009	0.005	0.762
MLF	0.473	0.169	-0.006	0.002	0.619
FAT	1.000	0.000	-0.009	0.009	1.000
CST	0.034	0.482	-0.009	-0.000	0.242

Table S4: Within-groups LI results. Statistically significant p-FDR values are highlighted in bold.

Tract	Group	p-value	effect-size	CI-lower	CI-upper	p-FDR
AF	HC	0.695	7.273	-0.003	0.002	0.834
AF	nvASD	0.557	10.752	-0.002	0.004	0.716
UF	HC	0.343	4.427	-0.006	0.002	0.515
UF	nvASD	0.492	11.068	-0.004	0.008	0.681
SLF_II	HC	0.004	0.316	-0.008	-0.002	**0.035**
SLF_II	nvASD	0.944	5.376	-0.007	0.007	0.944
SLF_III	HC	0.002	0.000	-0.014	-0.007	**0.035**
SLF_III	nvASD	0.010	0.949	-0.013	-0.002	**0.044**
ILF	HC	0.009	14.230	0.001	0.006	**0.044**
ILF	nvASD	0.049	14.863	0.001	0.012	0.110
IFO	HC	0.262	5.060	-0.005	0.002	0.429
IFO	nvASD	0.846	9.487	-0.005	0.008	0.944
MLF	HC	0.183	8.854	-0.002	0.008	0.330
MLF	nvASD	0.020	15.811	0.001	0.006	0.070
FAT	HC	0.049	14.863	0.000	0.011	0.110
FAT	nvASD	0.084	14.230	-0.000	0.011	0.168
CST	HC	0.027	1.897	-0.007	-0.001	0.082
CST	nvASD	0.922	9.171	-0.004	0.005	0.944

1.3 Radial Diffusivity (RD): Lateralization results

Table S5: Between-groups LI results. Statistically significant p-FDR values are highlighted in bold.

Tract	p-value	effect-size	CI-lower	CI-upper	p-FDR
AF	0.520	0.152	-0.006	0.002	0.669
UF	0.427	0.186	-0.010	0.005	0.641
SLF_II	0.015	0.541	-0.014	-0.002	0.132
SLF_III	0.247	0.270	-0.010	0.003	0.445
ILF	0.123	0.355	-0.011	0.002	0.369
IFO	0.684	0.101	-0.009	0.007	0.770
MLF	0.218	0.287	-0.007	0.002	0.445
FAT	0.971	0.017	-0.009	0.009	0.971
CST	0.063	0.423	-0.015	0.001	0.284

Table S6: Within-groups LI results. Statistically significant p-FDR values are highlighted in bold.

Tract	Group	p-value	effect-size	CI-lower	CI-upper	p-FDR
AF	HC	0.322	12.017	-0.002	0.004	0.414
AF	nvASD	0.059	10.119	-0.000	0.008	0.166
UF	HC	0.064	2.846	-0.010	0.001	0.166
UF	nvASD	0.441	3.795	-0.012	0.005	0.529
SLF_II	HC	0.009	0.000	-0.010	-0.004	**0.033**
SLF_II	nvASD	0.492	11.068	-0.005	0.008	0.554
SLF_III	HC	0.002	0.000	-0.018	-0.010	**0.012**
SLF_III	nvASD	0.002	0.000	-0.017	-0.006	**0.012**
ILF	HC	0.678	8.380	-0.004	0.005	0.695
ILF	nvASD	0.084	14.230	-0.001	0.010	0.168
IFO	HC	0.131	3.795	-0.006	0.001	0.196
IFO	nvASD	0.695	7.273	-0.008	0.005	0.695
MLF	HC	0.004	17.076	0.003	0.009	**0.018**
MLF	nvASD	0.002	17.393	0.005	0.012	**0.012**
FAT	HC	0.124	11.384	-0.003	0.014	0.196
FAT	nvASD	0.131	13.598	-0.002	0.011	0.196
CST	HC	0.084	3.162	-0.008	0.002	0.168
CST	nvASD	0.193	10.752	-0.002	0.014	0.267

1.4 Myelin Water Fraction (MWF): Lateralization results

Table S7: Between-groups LI results. Statistically significant p-FDR values are highlighted in bold.

Tract	p-value	effect-size	CI-lower	CI-upper	p-FDR
AF	0.063	0.423	-0.047	0.001	0.113
UF	0.015	0.541	-0.061	-0.005	0.066
SLF_II	0.353	0.220	-0.029	0.016	0.353
SLF_III	0.089	0.389	-0.051	0.006	0.134
ILF	0.165	0.321	-0.054	0.005	0.186
IFO	0.063	0.423	-0.048	0.003	0.113
MLF	0.143	0.338	-0.034	0.005	0.184
FAT	0.063	0.423	-0.062	0.002	0.113
CST	0.005	0.609	-0.032	-0.006	**0.047**

Table S8: Within-groups LI results. Statistically significant p-FDR values are highlighted in bold.

Tract	Group	p-value	effect-size	CI-lower	CI-upper	p-FDR
AF	HC	0.492	6.325	-0.027	0.012	0.681
AF	nvASD	0.064	14.546	-0.000	0.027	0.193
UF	HC	0.375	5.692	-0.038	0.012	0.614
UF	nvASD	0.014	16.128	0.006	0.039	0.062
SLF_II	HC	0.625	10.436	-0.016	0.024	0.750
SLF_II	nvASD	0.131	13.598	-0.003	0.024	0.262
SLF_III	HC	0.375	11.700	-0.015	0.034	0.614
SLF_III	nvASD	0.002	17.393	0.018	0.048	**0.018**
ILF	HC	0.922	9.171	-0.024	0.025	0.922
ILF	nvASD	0.020	15.811	0.006	0.047	0.070
IFO	HC	0.922	8.222	-0.024	0.016	0.922
IFO	nvASD	0.105	13.914	-0.006	0.037	0.237
MLF	HC	0.695	10.119	-0.014	0.020	0.782
MLF	nvASD	0.004	17.076	0.008	0.028	**0.023**
FAT	HC	0.432	6.008	-0.039	0.014	0.647
FAT	nvASD	0.084	14.230	-0.000	0.042	0.216
CST	HC	0.557	10.752	-0.009	0.013	0.716
CST	nvASD	0.002	17.393	0.014	0.030	**0.018**

1.5 T_2 relaxation time of intra- and extra-axonal water (T2IE): Lateralization results

Table S9: Between-groups LI results. Statistically significant p-FDR values are highlighted in bold.

Tract	p-value	effect-size	CI-lower	CI-upper	p-FDR
AF	0.075	0.406	-0.000	0.011	0.113
UF	0.052	0.439	-0.000	0.012	0.113
SLF_II	0.063	0.423	-0.000	0.008	0.113
SLF_III	0.052	0.439	-0.000	0.017	0.113
ILF	0.739	0.085	-0.003	0.006	0.739
IFO	0.075	0.406	-0.000	0.009	0.113
MLF	0.007	0.592	0.001	0.007	0.062
FAT	0.105	0.372	-0.003	0.014	0.118
CST	0.089	0.389	-0.001	0.008	0.115

Table S10: Within-groups LI results. Statistically significant p-FDR values are highlighted in bold.

Tract	Group	p-value	effect-size	CI-lower	CI-upper	p-FDR
AF	HC	0.846	9.487	-0.004	0.006	0.895
AF	nvASD	0.006	0.632	-0.006	-0.002	**0.018**
UF	HC	0.770	7.589	-0.007	0.005	0.866
UF	nvASD	0.010	0.949	-0.010	-0.003	**0.022**
SLF_II	HC	0.375	5.692	-0.004	0.003	0.614
SLF_II	nvASD	0.004	0.316	-0.006	-0.002	**0.018**
SLF_III	HC	0.770	7.589	-0.008	0.008	0.866
SLF_III	nvASD	0.004	0.316	-0.010	-0.005	**0.018**
ILF	HC	0.322	5.376	-0.007	0.002	0.580
ILF	nvASD	0.006	0.632	-0.006	-0.001	**0.018**
IFO	HC	0.695	10.119	-0.003	0.006	0.866
IFO	nvASD	0.004	0.316	-0.005	-0.001	**0.018**
MLF	HC	0.625	6.957	-0.003	0.004	0.865
MLF	nvASD	0.002	0.000	-0.007	-0.002	**0.018**
FAT	HC	0.492	11.068	-0.005	0.010	0.738
FAT	nvASD	0.160	4.111	-0.007	0.002	0.320
CST	HC	1.000	8.538	-0.004	0.003	1.000
CST	nvASD	0.010	0.949	-0.007	-0.001	**0.022**

2. Supplementary Figures for Quality Control: Contour plot for each segmented tract

All subjects were linearly registered to the MNI space. A contour plot shows the outer boundary of each segmented tract per brain hemisphere. The background image is the FA.

Segmented tracts

Legend

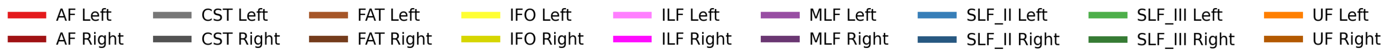

Control 01.png

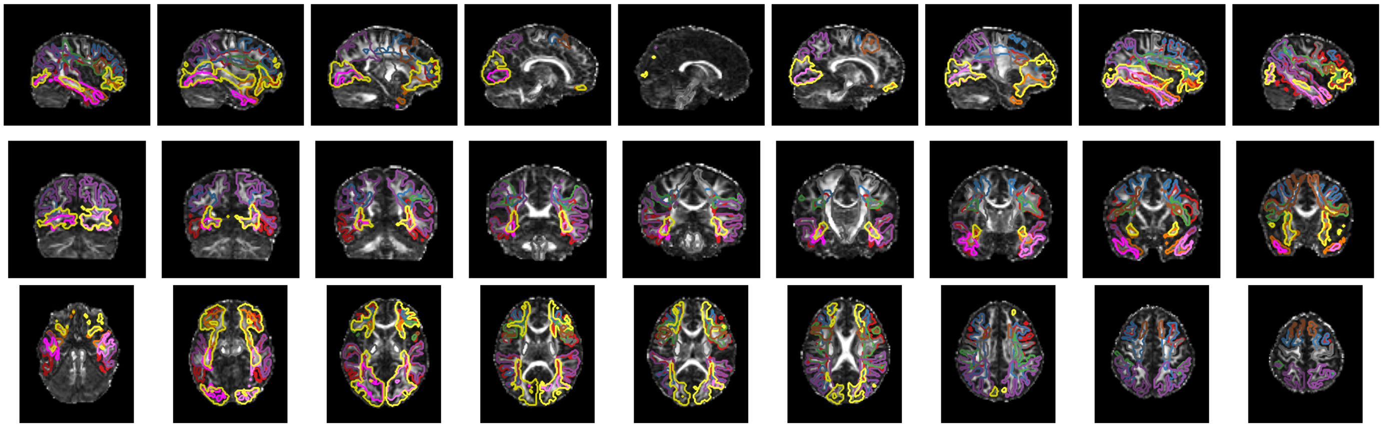

Control 02.png

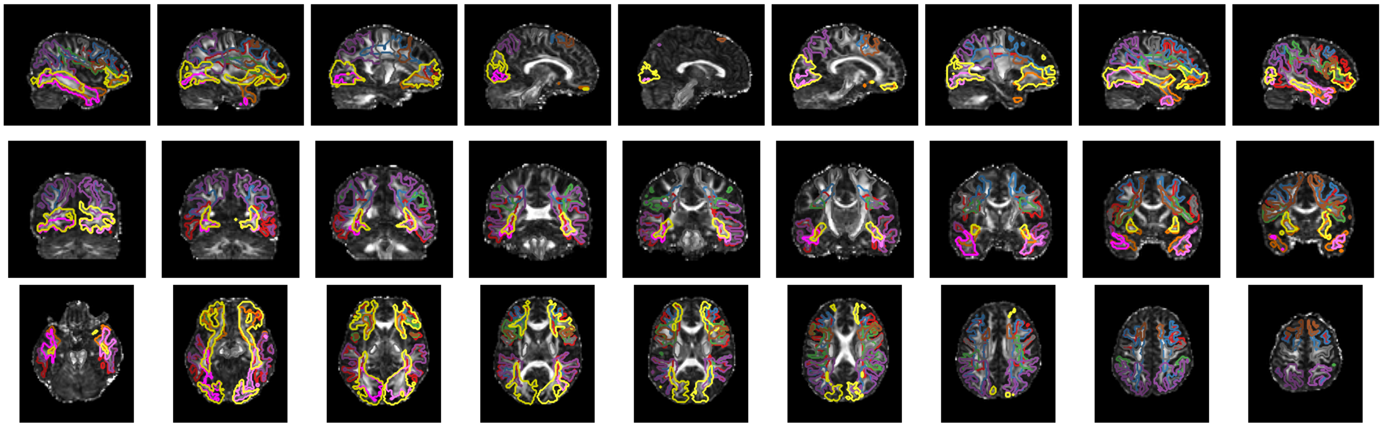

Control 03.png

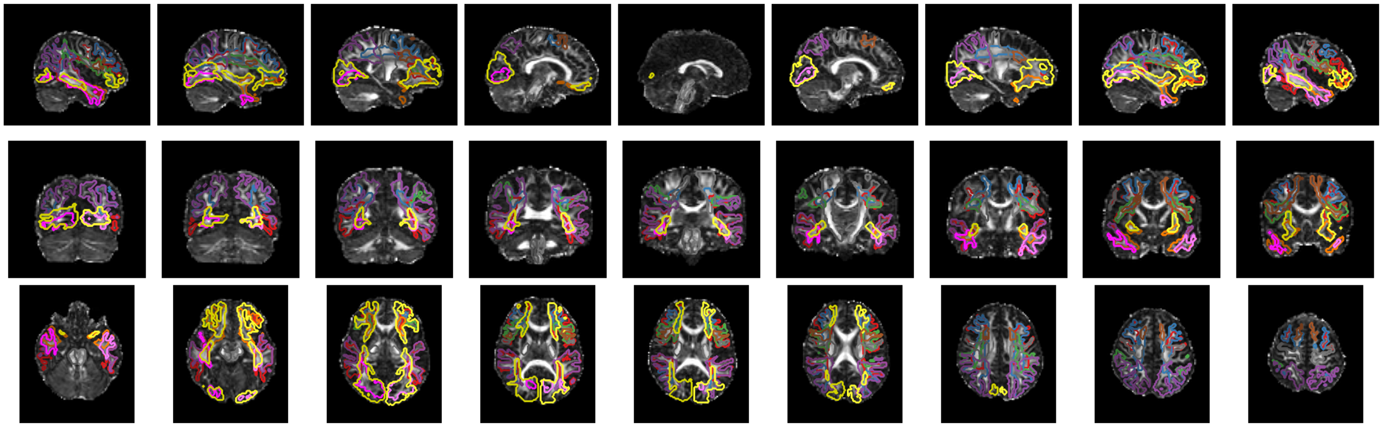

Control 04.png

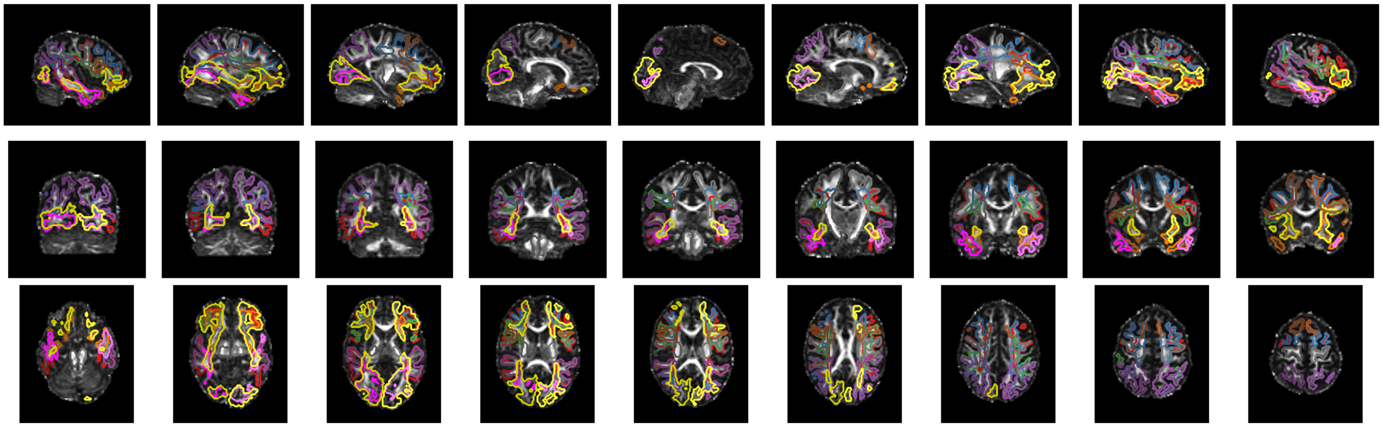

Control 05.png

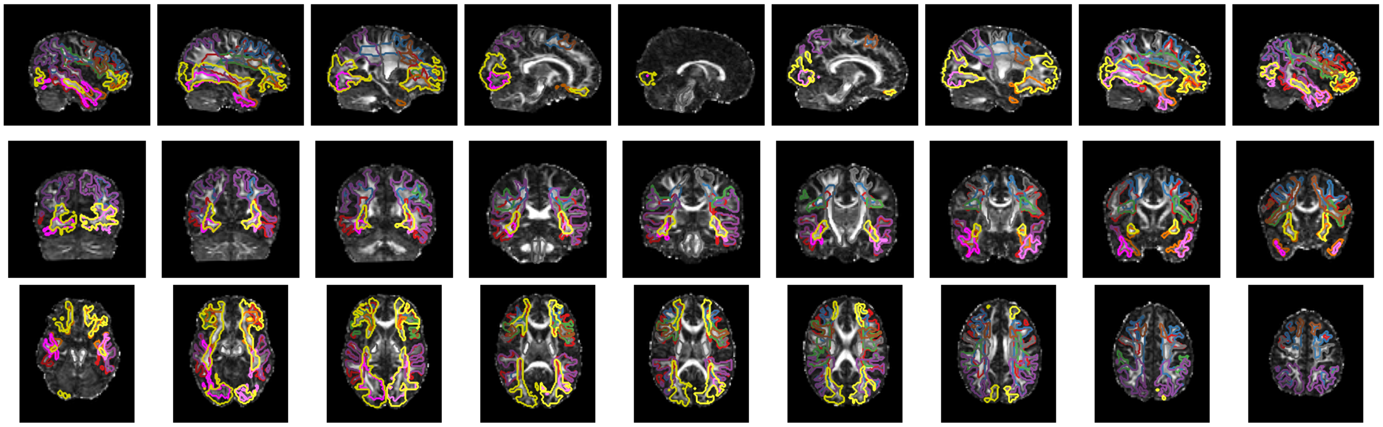

Control 06.png

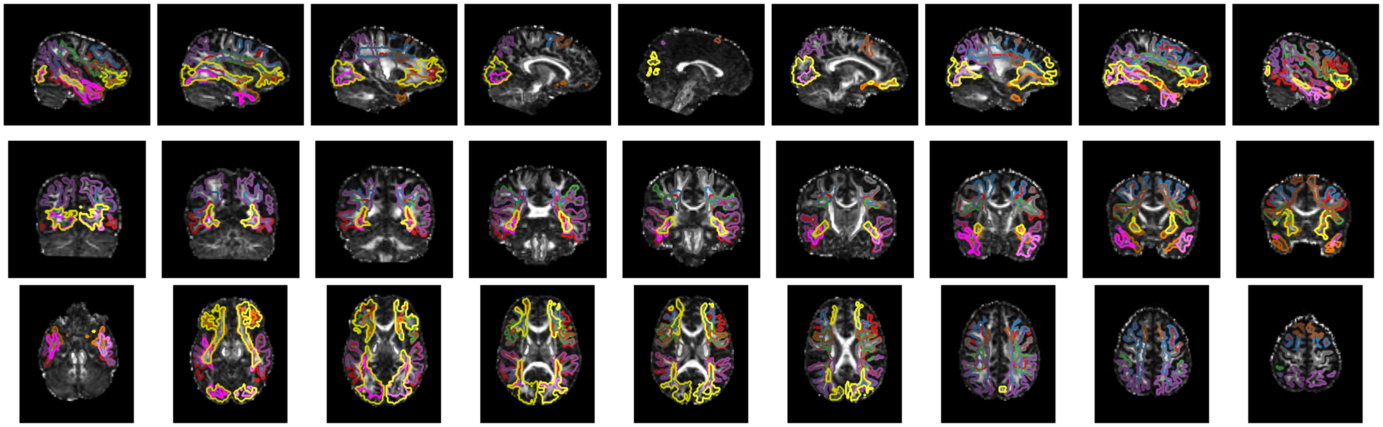

Control 07.png

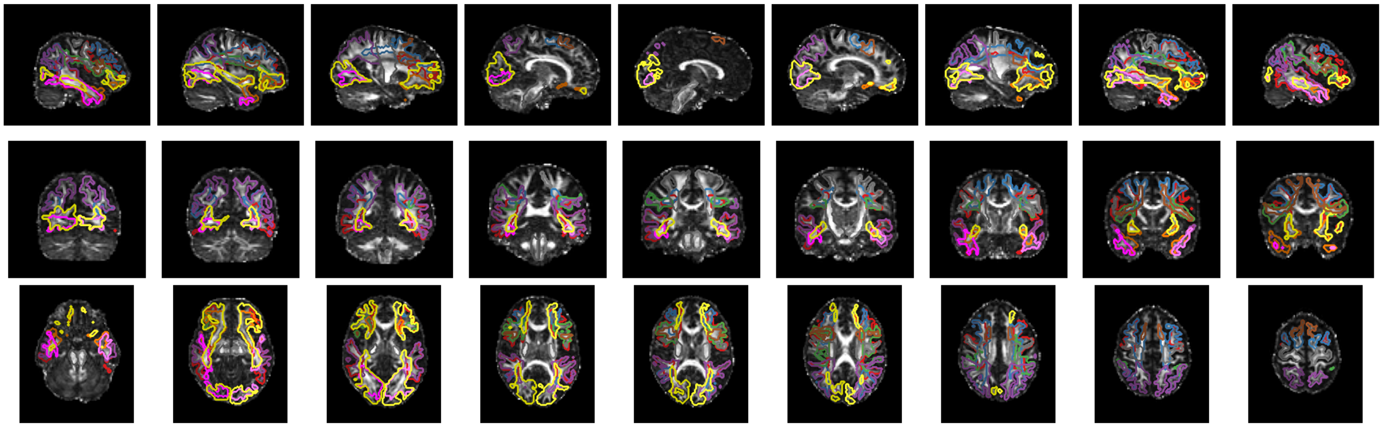

Control 08.png

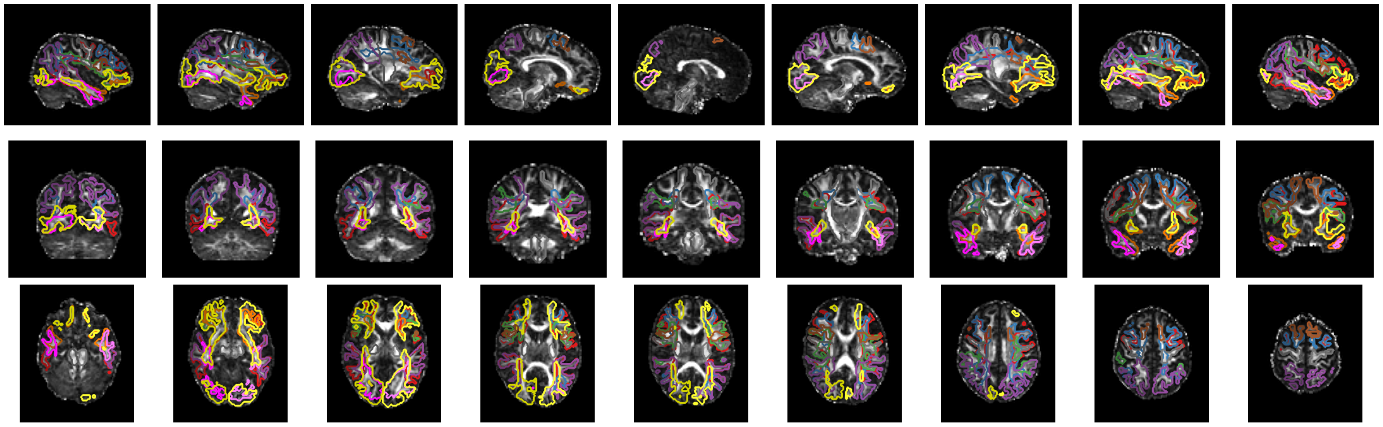

Control 09.png

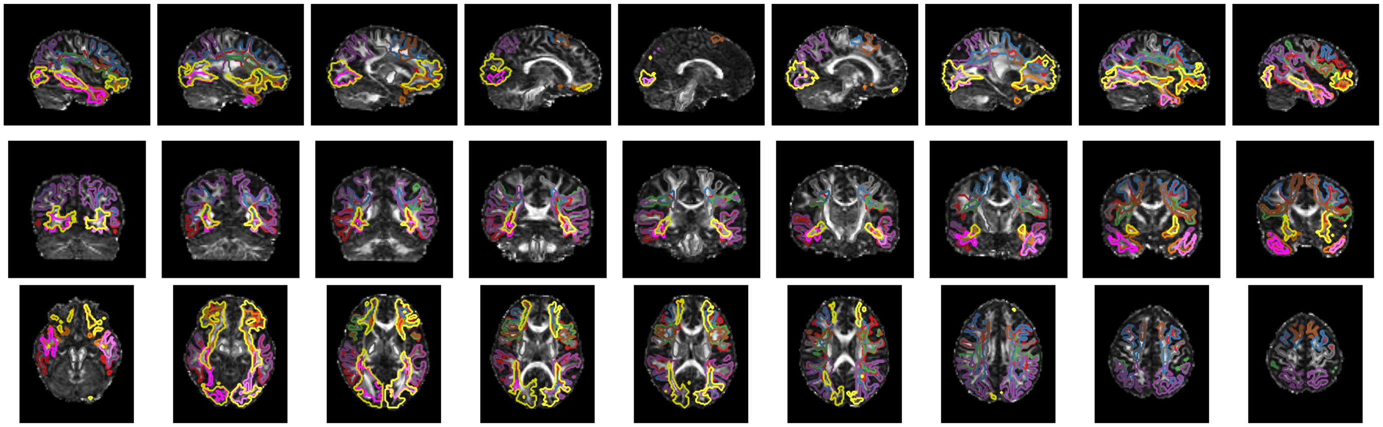

Control 10.png

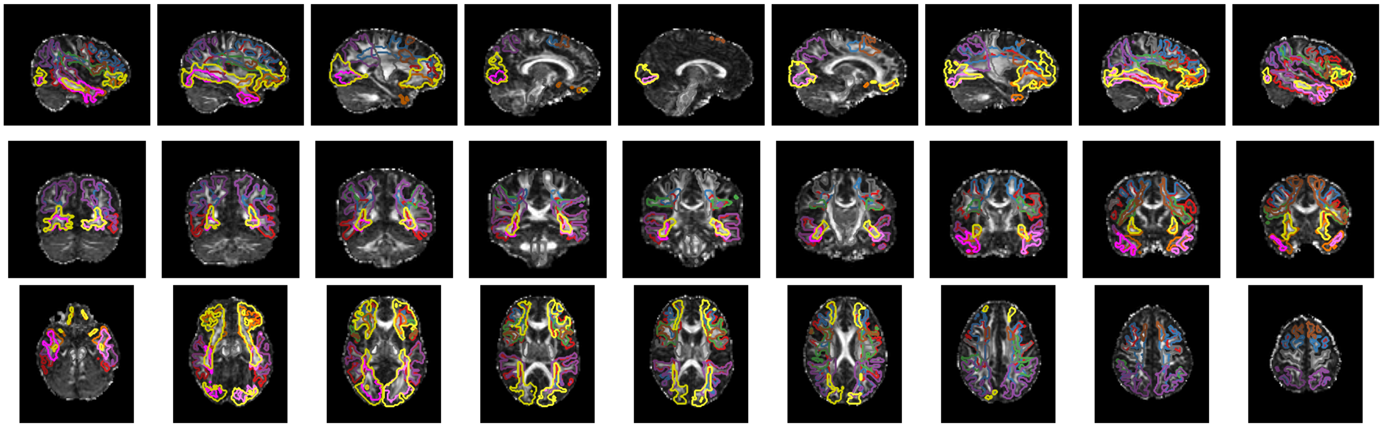

nvASD 01.png

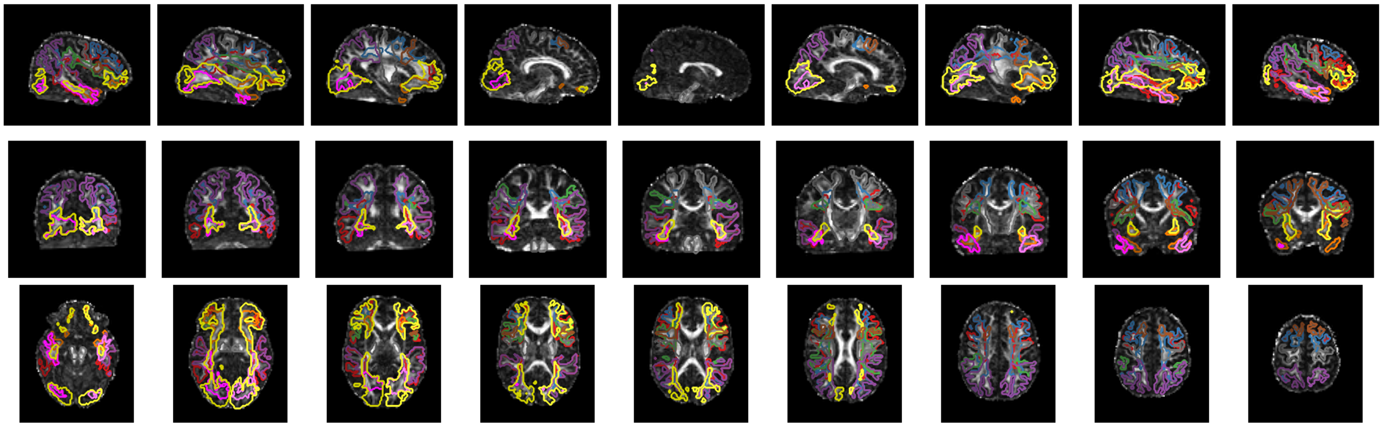

nvASD 02.png

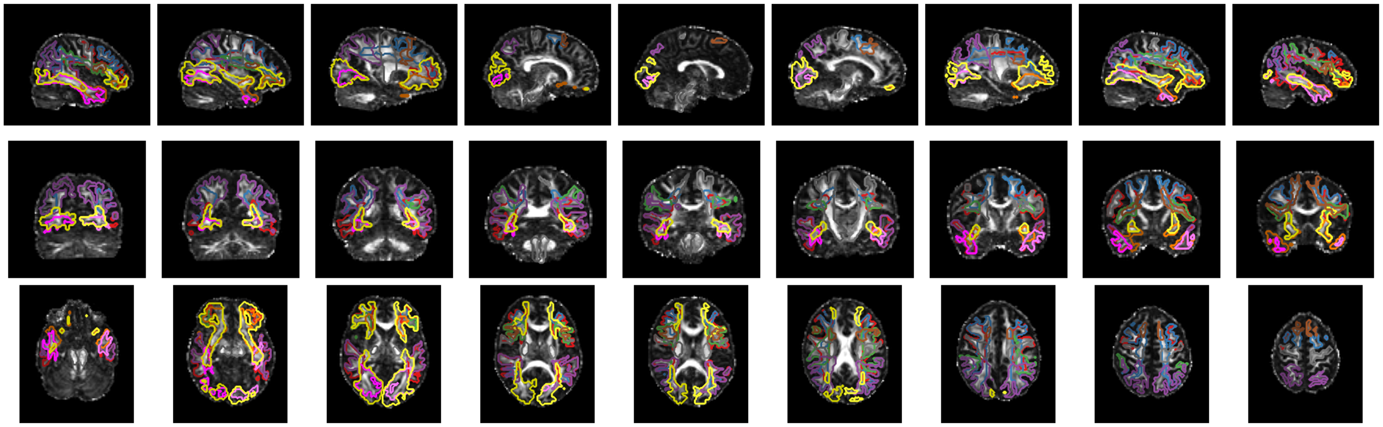

nvASD 03.png

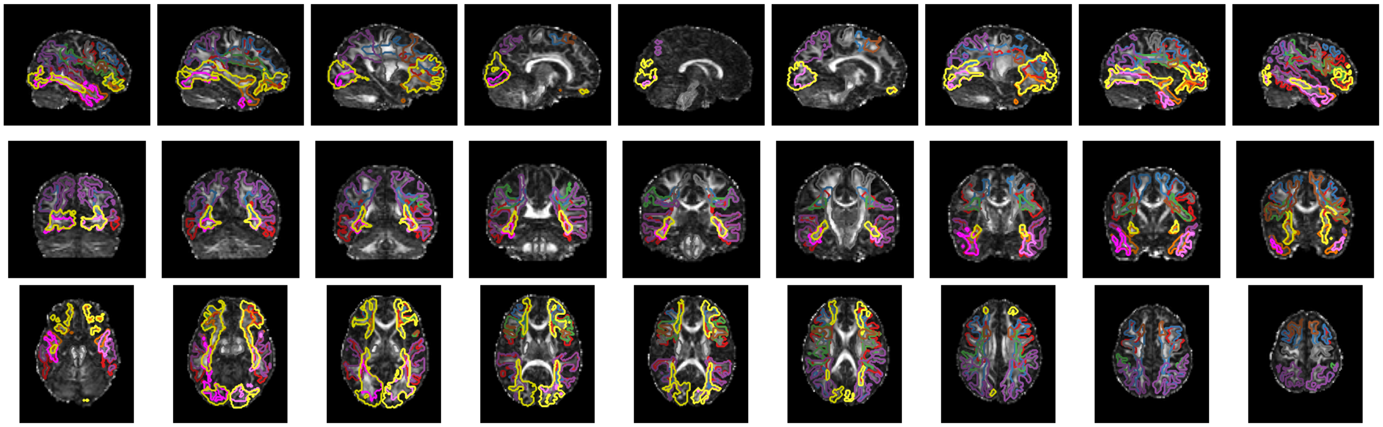

nvASD 04.png

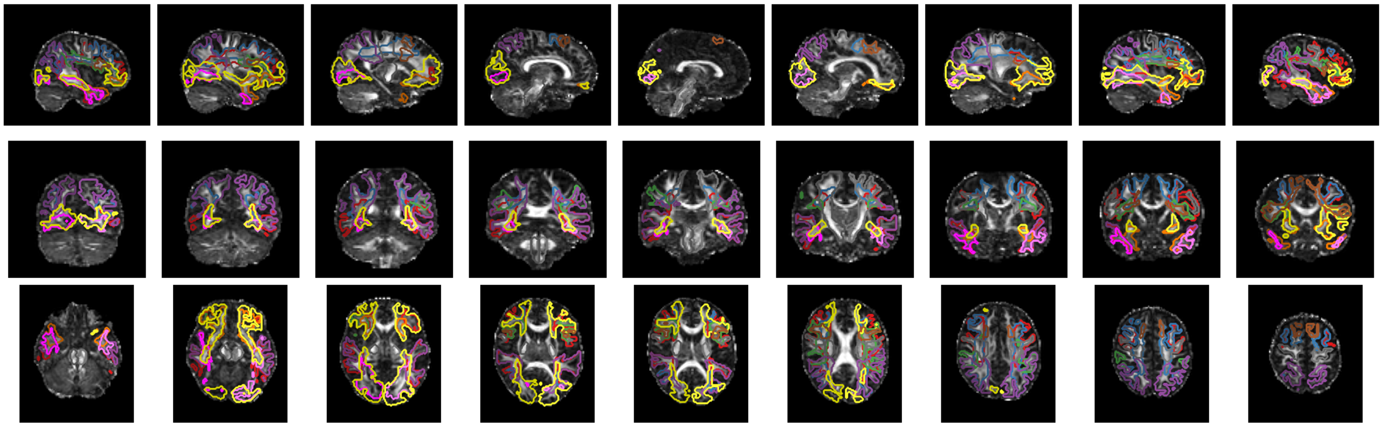

nvASD 05.png

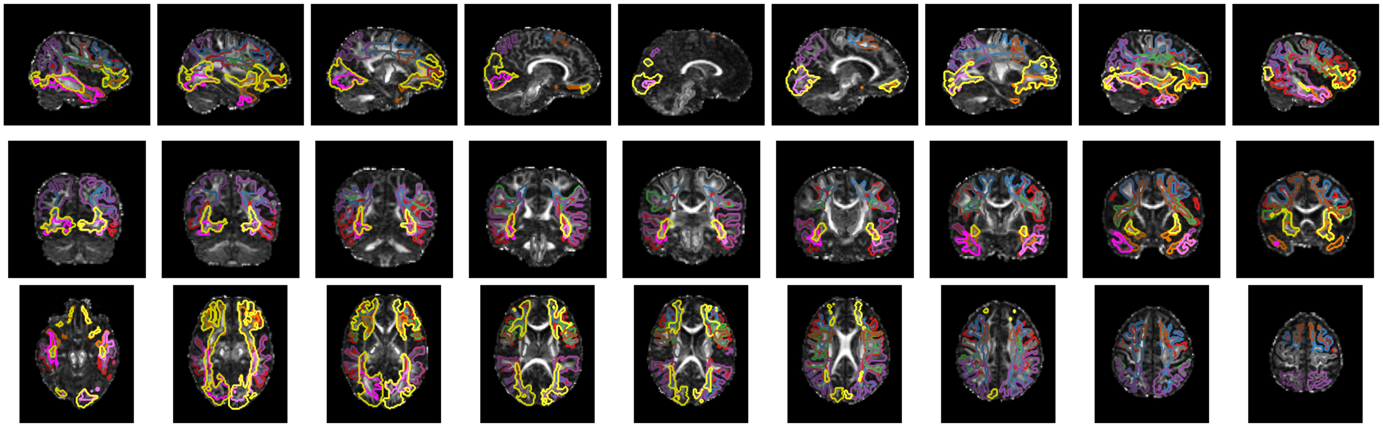

nvASD 06.png

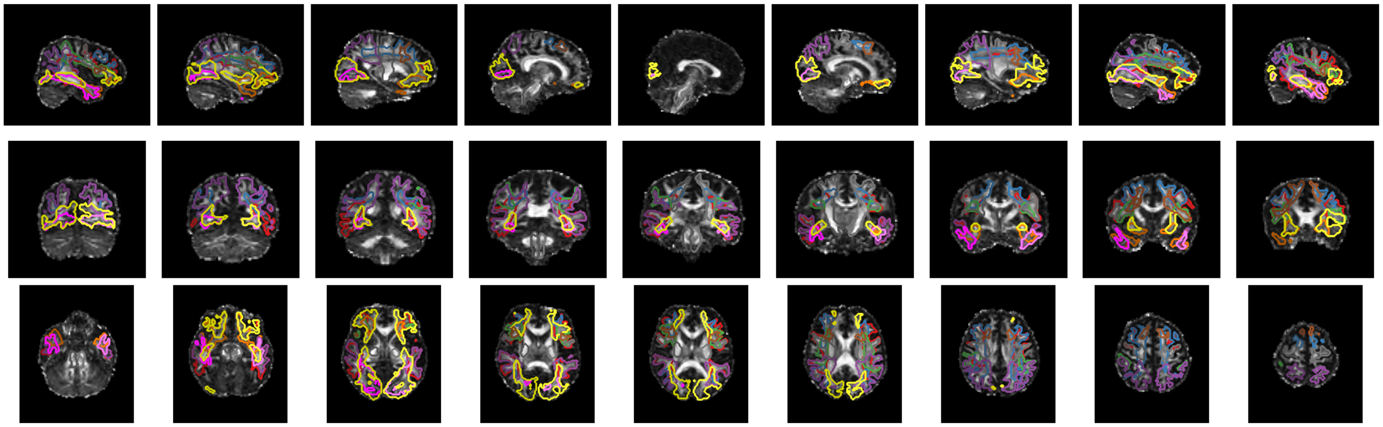

nvASD 07.png

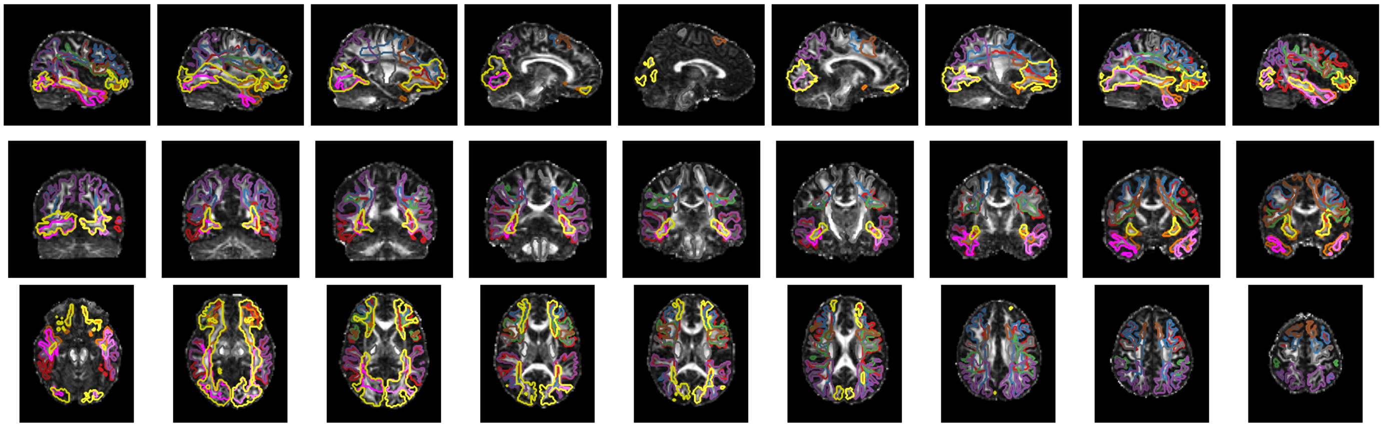

nvASD 08.png

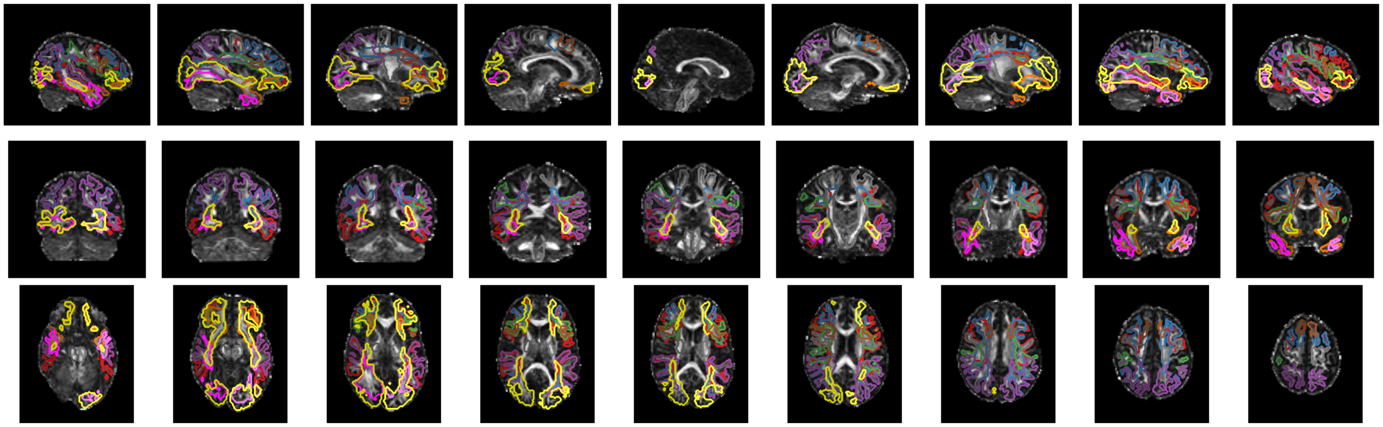

nvASD 09.png

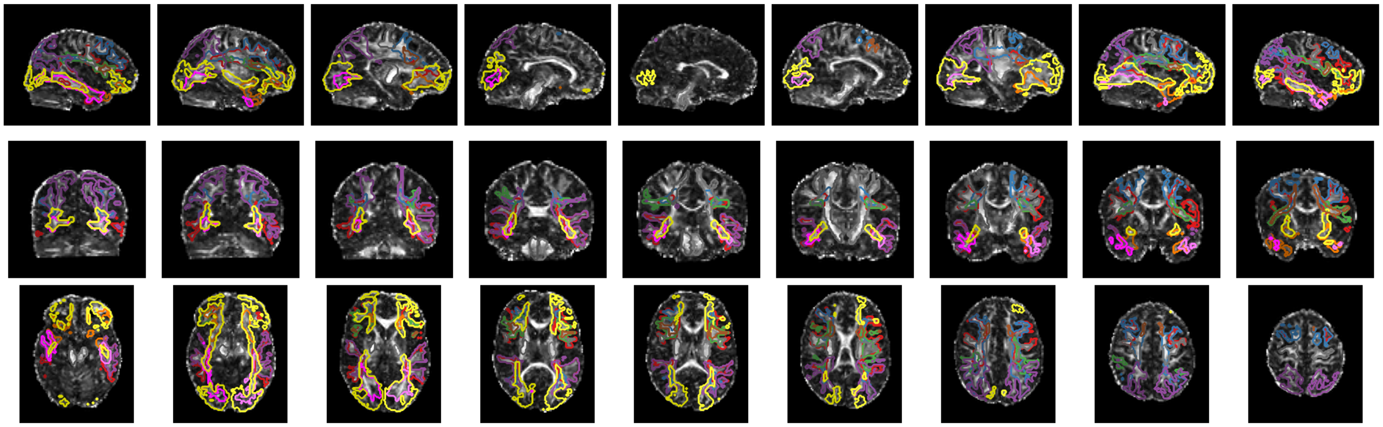

nvASD 10.png

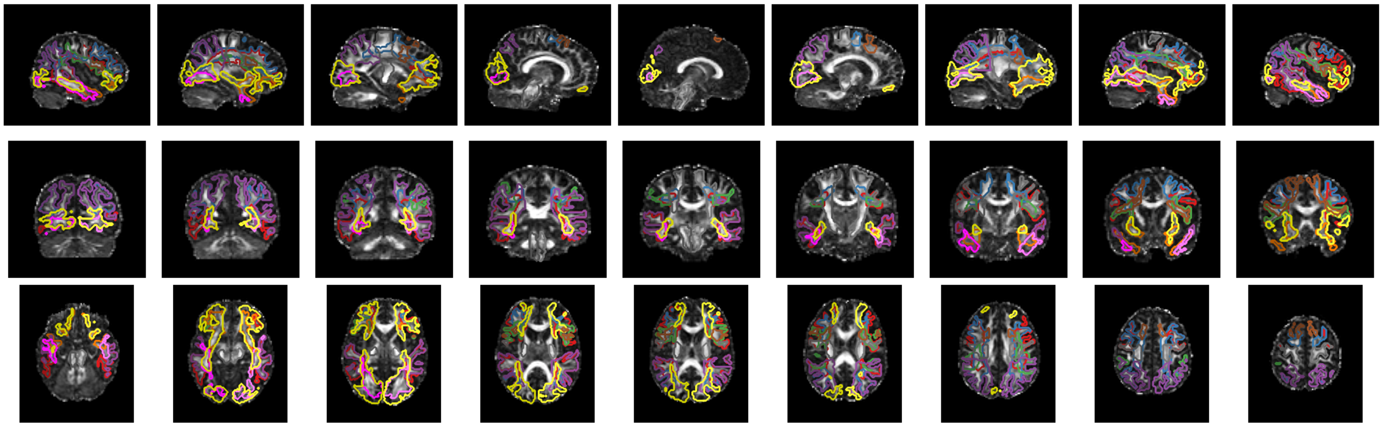

3. Results from the reanalysis

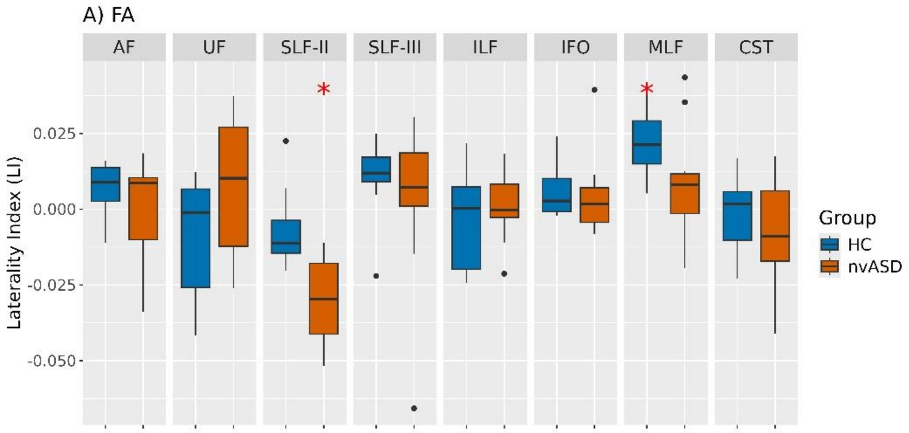

Supplementary Figure. Laterality index (*LI*) values derived from Fractional Anisotropy (FA) metric for healthy controls (HC) and individuals with nvASD across white matter tracts. Significant *LI* values ($LI \neq 0$) per tract, corrected for multiple comparisons, are marked with a red asterisk. No significant inter-group *LI* differences were found.
